# Gene Expression Network Analysis of Precursor Lesions in Familial Pancreatic Cancer

**DOI:** 10.1089/pancan.2020.0007

**Published:** 2020-08-05

**Authors:** Ming Tan, Ove B. Schaffalitzky de Muckadell, Maiken Thyregod Jøergensen

**Affiliations:** ^1^ Department of Medical Gastroenterology, Odense University Hospital, Odense, Denmark.; ^2^ Department of Clinical Research, University of Southern Denmark, Odense, Denmark.; ^3^ Odense Pancreas Center (OPAC), Odense University Hospital, Odense, Denmark.

**Keywords:** familial pancreatic cancer, gene expression, network-based analysis, precursor lesions, sporadic pancreatic cancer

## Abstract

**Purpose:** High-grade pancreatic intraepithelial neoplasia (PanIN) are aggressive premalignant lesions, associated with risk of progression to pancreatic ductal adenocarcinoma (PDAC). A depiction of co-dysregulated gene activity in high-grade familial pancreatic cancer (FPC)-related PanIN lesions may characterize the molecular events during the progression from familial PanIN to PDAC.

**Materials and Methods:** We performed weighted gene coexpression network analysis (WGCNA) to identify clusters of coexpressed genes associated with FPC-related PanIN lesions in 13 samples with PanIN-2/3 from FPC predisposed individuals, 6 samples with PDAC from sporadic pancreatic cancer (SPC) patients, and 4 samples of normal donor pancreatic tissue.

**Results:** WGCNA identified seven differentially expressed gene (DEG) modules and two commonly expressed gene (CEG) modules with significant enrichment for Gene Ontology (GO) terms in FPC and SPC, including three upregulated (*p* < 5e-05) and four downregulated (*p* < 6e-04) gene modules in FPC compared to SPC. Among the DEG modules, the upregulated modules include 14 significant genes (*p* < 1e-06): *ALOX12-AS1*, *BCL2L11*, *EHD4*, *C4B*, *BTN3A3*, *NDUFA11*, *RBM4B*, *MYOC*, *ZBTB47*, *TTTY15*, *NAPRT*, *LOC102606465*, *LOC100505711*, and *PTK2*. The downregulated modules include 170 genes (*p* < 1e-06), among them 13 highly significant genes (*p* < 1e-10): *COL10A1*, *SAMD9*, *PLPP4*, *COMP*, *POSTN*, *IGHV4–31*, *THBS2*, *MMP9*, *FNDC1*, *HOPX*, *TMEM200A*, *INHBA*, and *SULF1*. The DEG modules are enriched for GO terms related to mitochondrial structure and adenosine triphosphate metabolic processes, extracellular structure and binding properties, humoral and complement mediated immune response, ligand-gated ion channel activity, and transmembrane receptor activity. Among the CEG modules, *IL22RA1*, *DPEP1*, and *BCAT1* were found as highly connective hub genes associated with both FPC and SPC.

**Conclusion:** FPC-related PanIN lesions exhibit a common molecular basis with SPC as shown by gene network activities and commonly expressed high-connectivity hub genes. The differential molecular pathology of FPC and SPC involves multiple coexpressed gene clusters enriched for GO terms including extracellular activities and mitochondrion function.

## Introduction

Pancreatic cancer is among the deadliest cancer diagnoses worldwide, with a 5-year survival of just 9.3% in western populations.^1^ An estimated fraction of up to 10% of all pancreatic cancers is attributed to familial pancreatic cancer (FPC)—with members in affected families having a life-time risk of up to 39% of developing pancreatic ductal adenocarcinoma (PDAC).^2,3^ The greatest challenge in the treatment of pancreatic cancer is early diagnosis of the disease, enabling surgical treatment. A potential prospect in the treatment of PDAC is targeted treatment of cancer cells on a molecular level, intervening in different pathways of PDAC cell proliferation, growth, and tumor invasion.

Despite the large extent of genomic studies on FPC,^4,5^ little effort has been taken in examining the interactive nature in the genome in FPC studies using systems biology approaches such as the weighted gene coexpression network analysis (WGCNA).^6^ The application of WGCNA to identify gene expression networks potentially involved in crucial molecular pathways for progression of cancer is becoming an increasingly prevalent area in oncogenomic studies among different types of high-morbidity cancers, including breast cancer, colon cancer, gastric cancer, and pancreatic cancer.^7–10^ Implementation of WGCNA on genomic data on FPC could open new perspectives for the characterization of hereditary pancreatic cancer—identifying important pathways and hub genes associated with molecular mechanisms in the progression of FPC.

Individuals with known disposition for FPC are a high-risk group for developing premalignant pancreatic lesions with potential progression to PDAC. The process of progression from premalignant lesions to the formation of invasive PDAC is thought to take around 10 years.^11^ The most prominent precursor lesions, pancreatic intraepithelial neoplasia (PanIN), are present in around 82% of all PDAC cases,^12^ with high-grade PanINs (grade 2–3), in particular being prominently presented in FPC.^13^ High-grade PanINs are shown to have distinctively different molecular characteristics compared to non-PanIN-related PDACs.^14,15^ Gene expression profiling could, due to the distinctive gene expression activity found in high-grade PanIN lesions, prove highly relevant in the molecular characterization of PanINs in individuals from FPC families.^14^

A systems-level understanding of gene expression activity in FPC individuals with PanIN lesions and PDAC in sporadic pancreatic cancer (SPC) patients can help characterize the molecular events during the development and potential progression of PanIN to PDAC. Furthermore, Gene Ontology (GO) enrichment analysis on gene clusters with commonly expressed genes (CEGs) and differentially expressed genes (DEGs) can form an enhanced understanding of the cellular functionality of gene clusters expressed in PanIN and PDAC.

## Methods

We analyzed microarray gene expression data, retrieved from the public Gene Expression Omnibus (GEO) database (GEO series accession number: GSE43288).^16^ The retrieved data included normalized microarray gene expression data from 13 samples of PanIN lesions (stage 2–3) from FPC individuals, 6 samples of PDAC from SPC patients, and 4 samples of normal donor pancreata (used as controls) measured by Affymetrix GeneChip HG-U133 arrays. Details on microarray experiment and microarray data preprocessing can be found elsewhere.^17^ Following the sample quality assessment in the original publication, 2 samples of PDAC from SPC and a replicate of 1 normal specimen were dropped leaving a total of 20 samples for subsequent analysis.

IRB approval and participant consent were not applicable in this study.

### FPC family characteristics

The 13 samples from pancreatectomy specimens with PanIN lesions are derived from 4 different FPC families (family A, B, C, and X). The origin of each specimen are as following: one specimen derived from one patient of family A; three specimens from one patient of family B; two specimens from one patient of family C; and seven specimens from four patients of family X. Family characteristics of the FPC families are as presented in a previous publication.^17^

While family A, B, and C meet criteria for conventional FPC pedigrees (i.e., at least two affected first degree relatives with PDAC), family X is characterized by having a germline mutation in the palladin gene resulting in a highly penetrant, autosomal presentation of FPC (i.e., early onset of disease, preceded by endocrine and exocrine pancreatic insufficiency).^18^ Distribution of affected family members among the FPC families and age of onset of PDAC are as follows: family A (five cases of PDAC [age of onset: 64-NA]), family B (two cases of PDAC [age of onset: 53–66]), family C (four cases of PDAC [age of onset: 50–58]), and family X (nine cases of PDAC [age of onset: 28–57]).

### Data analysis

**FIG. 1.**
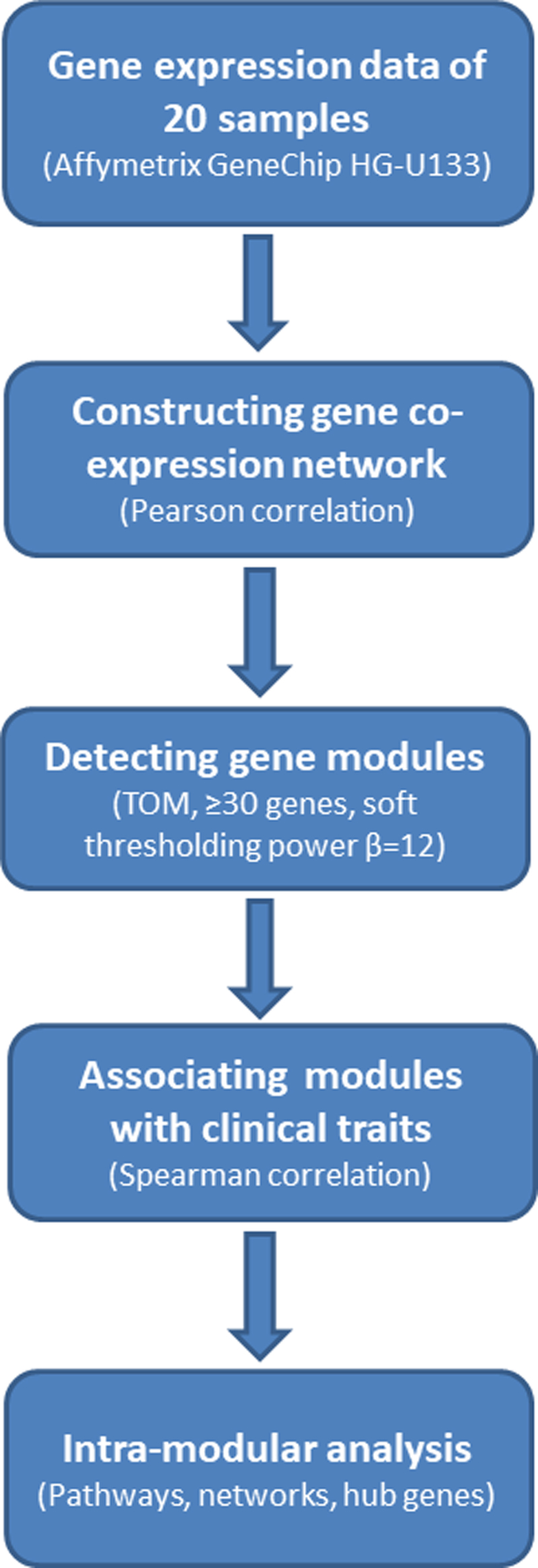
Flowchart illustrating the analytical steps of WGCNA. WGCNA, weighted gene coexpression network analysis.

We performed WGCNA using WGCNA (v1.47) package in R.^19^ In Figure 1, we show the flowchart for the analytical steps. WGCNA is a powerful network analysis tool that can be used to identify groups of highly correlated genes that co-occur across samples. In brief, WGCNA first constructed a matrix of adjacencies by calculating Pearson correlations in all pairs of genes across samples, which was then computed into a Topological Overlap Matrix (TOM). TOM is a metric of interconnectivity in all gene pairs and was used as input for hierarchical clustering analysis with a cluster of genes of high topological overlap defined as a module. For each module, a module eigengene (ME) was defined as the first principal component from PCA (principle component analysis) on the expression of all genes from the module, to be considered as a representation of the overall gene expression profiles in the module. For each gene in a module, a module membership (MM) was calculated by correlating the ME with its gene expression values to quantify how close the gene was to the given module. The association between each module and a clinical phenotype was assessed by Spearman correlation considering categorical phenotypes and sample relatedness. For each module, gene significance (GS, absolute Spearman correlation coefficient of single gene expression levels with a clinical trait) was plotted against MM (Pearson correlation coefficient of single gene expression levels with the ME) and their correlation calculated for intramodular analysis and screening hub genes with high MM and GS. At the end, interesting modules were analyzed for enrichment of GO terms and/or visualized for their networks and high connectivity hub genes using VisANT software.^20^

## Results

The microarray data contained expression profiles of 44,928 probes summarized into 19,529 genes by taking the mean expression of all probes of each gene. In Supplementary Figure S1, the samples are clustered by the expression profiles of all genes measured on the microarray. The mega cluster to the left represents the sporadic cancer samples; the mega cluster to the right represents the FPC samples from family X; the mega cluster in the middle consists of one subcluster of three normal tissue samples designated as N and six FPC samples from families A, B, and C denoted as Non-X. The figure suggests that the clinical features in the collected samples can be characterized by the measured gene expression profiles.

**FIG. 2.**
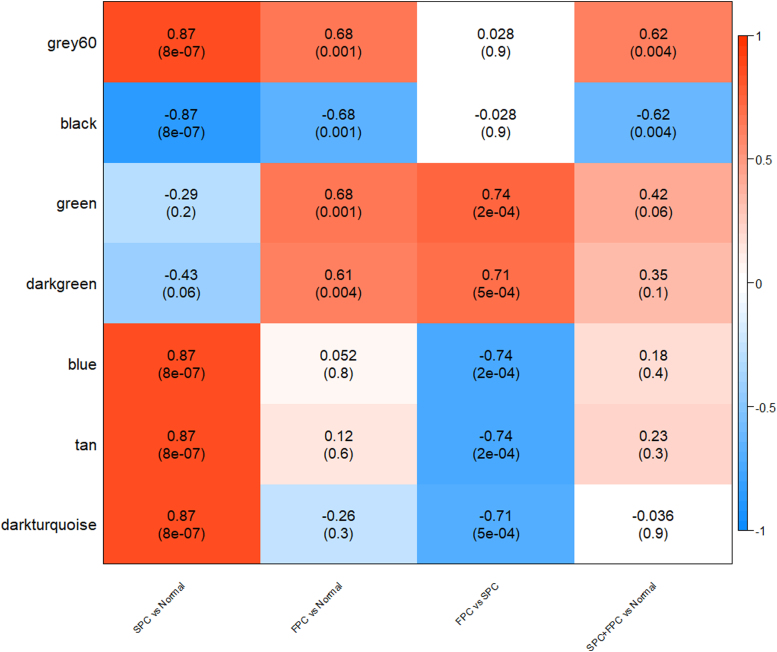
Correlation coefficient and *p*-value (in parenthesis) for the association of major gene modules represented by a module eigengene in each row indicated in the left with one clinical feature in each column indicated in the bottom. The color bar to the right indicates magnitude and direction of correlation. The deeper the red or blue, the higher the positive or negative correlation between the module eigengene and a clinical feature.

Following the analytical steps as illustrated in Figure 1 and described in the Methods section, we first clustered all genes—based on topological overlaps, into different modules (gene clusters) consisting of genes that are highly correlated in their expression levels. With minimum size of 30 genes and a soft thresholding power of 12 (Supplementary Fig. S2), 27 modules were detected (Supplementary Fig. S3) and analyzed for their associations with clinical features (e.g., SPC, FPC types, and combination of them) (Supplementary Fig. S4). Figure 2 presents the level and significance for the correlation between clinical feature in columns and the expression metric of major modules summarized as a ME in rows. The deeper the red or blue, the higher the positive or negative correlation between the ME and a trait. The figure reveals that there are modules or gene clusters highly significantly regulated in association with different clinical features.

The correlations in Figure 2 and Supplementary Figure S4 are also supported by plotting GS against MM in each module for SPC in Supplementary Figure S5, FPC in Supplementary Figure S6, and FPC versus SPC in Supplementary Figure S7 with each dot representing a gene and those plotted to the top right of each plot representing high connectivity to other genes and high correlation with clinical features (the hub genes), reconfirming the high associations between gene modules with FPC (including black, brown, green, gray60) and SPC (including black, blue, gray60).

### DEG modules

Correlation analysis identified DEG modules—with four upregulated modules (orange, green, light green, and dark green, *p* < 5e-04) and four downregulated modules (blue, cyan, tan, and dark turquoise, *p* < 5e-04) in the FPC group compared to the SPC group (Supplementary Fig. S4). The upregulated gene modules include 14 significant genes (*p* < 1e-06) consisting of *ALOX12-AS1*, *BCL2L11*, *EHD4*, *C4B/C4A*, *BTN3A3*, *NDUFA11*, *RBM4B*, *MYOC*, *ZBTB47*, *TTTY15*, *NAPRT*, *LOC102606465*, *LOC100505711*, and *PTK2.* The downregulated gene modules include 169 significant genes (*p* < 1e-06), among them 13 highly significant genes (*p* < 1e-10) consisting of *COL10A1*, *SAMD9*, *PLPP4*, *COMP*, *POSTN*, *IGHV4–31*, *THBS2*, *MMP9*, *FNDC1*, *HOPX*, *TMEM200A*, *INHBA*, and *SULF1.*

**FIG. 3.**
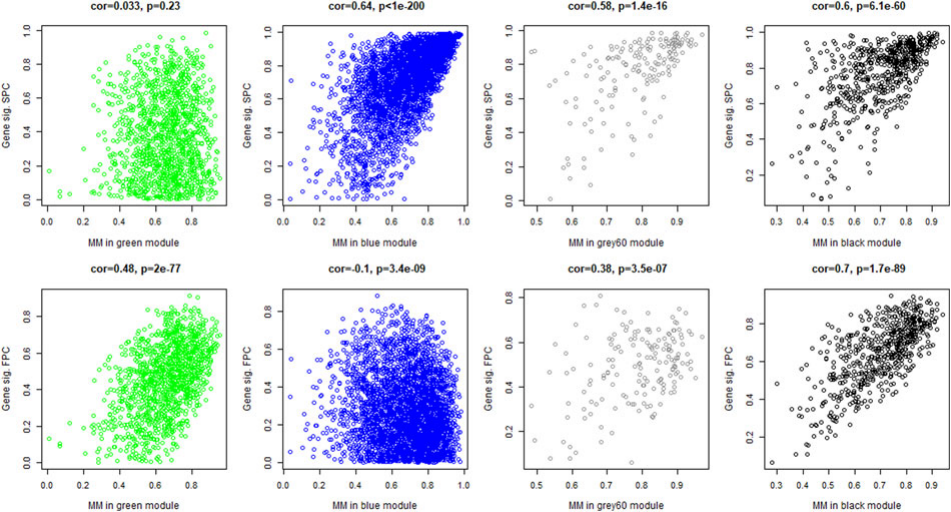
MM versus gene significance for top gene modules significant for both SPC and FPC (gray60 and black modules), for FPC (green module) and for SPC (blue module) only. FPC, familial pancreatic cancer; MM, module membership; SPC, sporadic pancreatic cancer.

In Figure 3, GS of each gene is plotted against its MM within a module for the green module upregulated in FPC and the blue module upregulated in SPC. The GS versus MM plots for direct differential expression between FPC and SPC for all modules are presented in Supplementary Figure S7. Enrichments for GO terms among different modules are depicted in Supplementary Table S1. The upregulated *green* module is significantly enriched for GO terms functionally related to receptor activity, ligand-gated ion channel activity, and transmembrane signaling receptor activity. The *orange, dark green* and *light green* modules are not significantly enriched for any GO terms (*p* > 0.05). The downregulated modules include *blue*, *cyan*, and *dark turquoise*, and are significantly enriched for GO terms functionally related to *blue module*: extracellular structure organization, extracellular matrix (ECM) organization, vesicle, focal adhesion, cell-substrate adherens junction, collagen binding, ECM, adherens junction, and extracellular vesicle; *cyan module*: mitochondrial inner membrane, organelle inner membrane, mitochondrial envelope, mitochondrial membrane, adenosine triphosphate (ATP) metabolic process, mitochondrion, mitochondrial ATP synthesis coupled electron transport, oxidative phosphorylation, mitochondrial ATP synthesis coupled proton transport, and hydrogen ion transmembrane transport; and *dark turquoise module*: antigen binding, complement activation, classical pathway, humoral immune response mediated by circulating immunoglobulin, phagocytosis, recognition, complement activation, and humoral immune response. The *brown* module, although significantly downexpressed, is not significantly enriched for any GO terms (*p* > 0.05).

### CEG modules

We identified CEG modules shared by both FPC and SPC—including one commonly upregulated module (*gray60*, *p* < 4e-03) and one commonly downregulated module (*black*, *p* < 4e-03) compared with normal pancreatic tissue (Fig. 2). Figure 3 plots GS against MM for gray60 and black modules showing significant correlation in both SPC and FPC. The commonly upregulated module, *gray60*, includes 69 highly significant genes (*p* < 1e-06) with seven top significant genes (*p* < 1e-12) consisting of: *EMP1*, *RND3*, *C11orf96*, *BNIP2*, *SLC25A5*, *CAV1*, and *GNAI1.* In the commonly downregulated module, *black*, 214 genes are differentially expressed in comparison with normal pancreatic tissue (*p* < 1e-06); among them, 15 are top significant genes (*p* < 1e-12) consisting of *UTP14A*, *PGM3*, *SLC1A4*, *PHGDH*, *MRM3*, *GMPPA*, *SNORD14D*, *NSA2*, *NUBP1*, *CCT4*, *IGBP1*, *FMOD*, *AADAC*, *ASNS*, and *LANCL2*.

In Supplementary Table S1, significance of enriched GO terms is tested for each module. The *black* module is significantly enriched for 10 GO terms (adjusted *p*-value <3.78e-05) involving endoplasmic reticulum (ER), protein targeting to endoplasmic reticulum, signal recognition particle-dependent (SRP-dependent) cotranslational protein targeting to membrane, *etc.* The *gray60* module is enriched for one GO term with borderline significance (adjusted *p*: 0.061) involving elastic fibers in ECM of connective tissue (Supplementary Table S1).

### Hub genes in CEG module

**FIG. 4.**
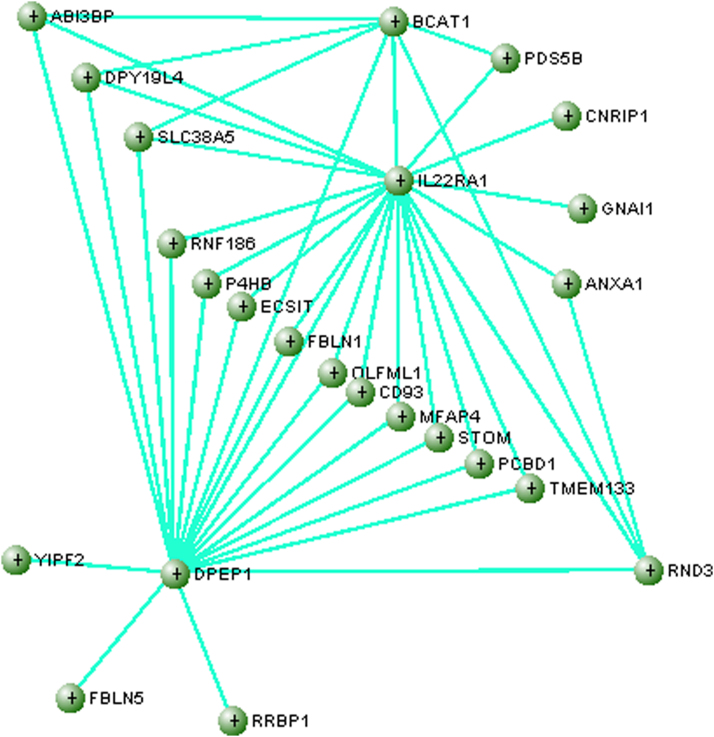
A novel gene expression network identified for shared coexpression patterns by SPC and FPC in gene module gray60. Three high connectivity genes are shown, *IL22RA1*, *DPEP1*, and *BCAT1*.

Visualization of the biological networks of the *gray60* module using VisANT software identified an interactive network with highly connective hub genes: *IL22RA1*, *DPEP1*, and *BCAT1* (with minimum number of connections of 7) as illustrated in Figure 4. The figure reveals the central role of the hub genes in coordinating the activities of other connected genes in the expression network.

## Discussion

While a published single-gene-based analysis on the same data identified 76 commonly dysregulated genes from normal pancreas to PanINs and PDAC,^17^ the present study is the first to apply the network-based WGCNA on FPC patients with PanIN-2/3 and SPC patients—identifying gene clusters and hub genes coregulated during the formation of PanIN and PDAC. Likewise, the present study is the first to directly compare GO terms on FPC individuals with PanIN-2/3 lesions to PDAC in SPC patients, characterizing the cellular functions of gene clusters with DEGs and CEGs between the two patient groups.

### DEG modules

In our analysis, we found gene modules with significant DEGs between patients with PanIN-2/3 lesions from FPC families and patients with SPC. The DEG modules were defined by three modules with significantly increased expression, and four modules with significantly decreased expression in FPC patients with PanIN-2/3 compared to SPC patients. The DEG modules with increased expression included genes functionally related to GO terms: receptor activity, ligand-gated ion channel activity, and transmembrane signaling receptor activity. DEG modules with decreased activity included genes functionally related to GO terms: mitochondrial structure and ATP metabolic processes, extracellular structure and binding properties, humoral and complement-mediated immune response, ligand-gated ion channel activity, and transmembrane receptor activity.

Due to the high metabolic ATP demands of cancer cells, sufficient mitochondrial and glycolytic ATP synthesis is pivotal to maintain cancer cell growth and survival. In the setting of pancreatic cancer, PDAC cells are shown to be solely vulnerable to cell death in the event of depleted glycolysis, but not mitochondrial ATP depletion—suggesting glycolytic ATP production to be more essential in PDACs compared to mitochondrial ATP synthesis.^21^ This correlates with present findings of decreased gene expression among genes coding for mitochondrial structure and mitochondrial ATP synthesis in FPC-related PanIN-2/3. These findings may underline the importance of aerobic glycolysis for ATP in PDAC—including a seeming shift from mitochondrial oxidative phosphorylation to glycolytic ATP production as the main energy source as described in the Warburg effect.^22–24^

It is worth mentioning that decreased expression in gene modules containing said genes related to mitochondrial structure and function was found in both the FPC and SPC patient groups—although with greater decrease in the FPC groups, despite that these patients only presented with PanIN-2/3 precursor lesions in comparison to the SPC group with invasive PDAC. Our findings suggest that the decreased expression of genes related to mitochondrial functions is more prominent in FPC-related PanIN than in PDAC in SPC patients. It would be of specific interest to examine in future settings, whether this difference in gene expression is due to the differential nature of premalignant PanIN compared to fulminant PDAC, or is explained by a difference between hereditary and sporadic disease.

Another downregulated gene module in FPC-related PanIN-2/3 was enriched for GO terms in extracellular structure organization and ECM organization. The ECM is known to be largely involved in functions related to proliferation and antiapoptosis in cancer cells, as well as cell migration and adhesion.^25–27^ When assessing the expression of the gene module involved in ECM functions in SPC alone, the DEGs have a significantly increased activity compared with normal controls, concordantly FPC patients with PanIN-2/3 lesions do not express a significantly increased activity in the same gene cluster—depicting a lack of the same amount of increase in ECM-related cell activity in premalignant lesions compared with invasive cancerous lesions, consistent with current knowledge.^28–30^ Our findings reaffirm that cells in precursor lesions do not express conformational and functional changes in the ECM compared to fulminant malignancy.

Gene expression activity was also significantly decreased among patients with FPC-related PanIN-2/3 compared to patients with SPC, in a gene module associated with complement activation and humoral immune response. Again, when examining gene activity in the particular module for SPC alone, gene expression is significantly increased when compared with normal controls, while activity was nondifferent in the FPC-related PanIN group compared with controls. Immune response is shown to play a complex role in the microenvironment of PDAC—with selected immune cells being suppressed in the setting of PDAC, while other cancer progressing immune cells are upregulated.^31,32^ Our findings support the increased immunologic activity in PDAC, while showing a lack of the same response in familial pancreatic precursor lesions—indicating that immune activity cannot be used as a marker for premalignant pancreatic lesions, unlike suggested among other types of cancers, including digestive tract carcinomas.^33^

The increased gene activity among genes responsible for ligand-gated ion channel activity and transmembrane receptor activity in the FPC group compared to SPC is quite interesting, as both mechanisms are involved in cellular processes associated with cell signaling, stimulation, growth, and proliferation—through ligand-gated ion channels and neurotransmitter signaling.^34,35^ The ligand-gated ion channels include subclasses such as the ATP-gated ion channels, activated through purinergic receptors, and transient receptor potential channels, whose signaling are involved in cell proliferation and mediation of immunological responses in the tumor microenvironment of PDAC cells.^35–39^ Another branch of ligand-gated ion channels are composed of neurotransmitter gated ion channels, including nicotinic acetylcholine receptor-based ion channels, and G-protein linked ion-channels. The suggestion that neurotransmitters may play a role in the progression of PDAC has previously been contemplated and supported.^40–43^ Interestingly, the gene module was only significantly upregulated in patients with FPC-related PanINs compared with normal controls, while gene activity was nondifferent in SPC patients—suggesting that ligand-gated ion channel activity to be more pronounced in pancreatic precursor lesions compared to PDAC. In consistence with present findings, the expression of a glutamate ionotropic receptor in ion channel was stepwise increased along with progression in premalignant PanIN 1–3 lesions, while being downregulated in PDAC compared to normal tissue.^44^

The present findings accentuate the importance of cell signaling—particularly through ligand-gated ion channels, including purinergic and glutamate receptor signaling, in the setting of PanIN. The upregulation of glutamate receptors in PanIN compared to PDAC suggests that glutamine receptor expression may be particularly involved in malignant transformation during progression of pancreatic precursor lesions to cancer.

It is necessary to mention that, although the two groups of FPC samples (X and non-X) in Supplementary Figure S4 display mostly similar patterns of module-trait correlation, the blue module is significantly upregulated in X while significantly downregulated in non-X samples. Overexpression of the blue module significantly implicated extracellular structure/matrix organizations could be a molecular feature of the X samples characterized by mutation in *paladin* gene, which encodes a component of the cytoskeleton as part of a complex important for cell structure and mobility. The differential regulation of the blue module in X and non-X samples represents the heterogeneous molecular pathology or microenvironment of FPC-related PanINs.

### CEG modules

The SPC and FPC groups shared two CEG modules, with one upregulated and one downregulated module. The CEG module related to ER protein targeting and localization was significantly downregulated in both SPC and FPC—accentuating the mechanisms underlying dysregulation in protein synthesis, transport, and protein folding during formation of premalignant and malignant pancreatic lesions.^45^ During cancer formation, the ER is known to be prone to a stress response caused by protein overexpression and accumulation of misfolded proteins in the ER—inducing the unfolded protein response (UPR) to reestablish ER homeostasis.^46^ Prolonged UPR signaling is shown to promote cell proliferation leading to further ER stress and activation of proangiogenic factors in response to hypoxia, finally resulting in the establishment of tumor microenvironment, supporting malignant cell formation and survival.^46^ Findings of the present study support previous implications suggesting endoplasmic sites of protein misfolding as potential targets in cancer therapy—targeting mediators of the UPR during ER stress.^47–49^

The upregulated CEG module between FPC and SPC is functionally related to elastic fibers in the ECM of connective tissue. Elastic fibers along with collagen fibers are the two main components of the ECM, and during development of PDAC elastosis of the ECM is a well-known phenomenon with vascular and ductal elastosis being highly prominent in tumor tissue in PDAC.^50^

### High-connectivity hub genes

The three high-connectivity hub genes presented in Figure 4 are previously shown to be involved in key features during the formation and progression of pancreatic cancer. In a previous study, it was shown that the Interleukin 22 receptor, alpha 1 (IL22RA1), encoded by the *IL22RA1* gene, is significantly upregulated in PDAC.^51^ Stimulation of the IL22R1A receptor by its major ligand IL-22, an interleukin expressed by immune cells, promotes cancer cell stemness, tumor cell proliferation, and higher degree of malignancy.^51–53^ It had previously been suggested that *IL22RA1* expression might be used as an independent prognostic marker in PDAC.^54^ In a contradicting manner, the expression of *IL22RA1* was found to be decreased in microarray data of both FPC and SPC—although as addressed in a previous study, this is likely due to the loss of pancreatic islet cells in the samples used for expression analysis, as *IL22RA1* is mainly expressed in Langerhans cells.^17,55^ Indeed, expression of *IL22RA1* was shown to be upregulated among DEGs in a dataset containing 90 PDACs.^56^ The identification of *IL22RA1* as a significant hub gene in the present study reaffirms the importance of immune-mediated pathways in the progression of cancers, as well as PDAC in particular.

We also identified *DPEP1* as a highly interconnected hub gene commonly expressed among both FPC and SPC. The cellular function of the Dipeptidase 1 enzyme (DPEP1) is the degradation of glutathione, an important antioxidant for cellular protection of pathological oxidative stress.^57^ The role of *DPEP1* during cancer tumorigenesis is described as somewhat heterogeneous—with studies showing an upregulation of *DPEP1* in colorectal cancer (CRC), functioning as a reverse prognostic marker in CRC, while expression of *DPEP1* is shown to be downregulated in the setting of PDAC and breast cancer, prompting it to have a tumorigenesis-inhibitory function in these settings.^58–61^ In the present study, expression of *DPEP1* was shown to be significantly downregulated in both FPC and SPC, compared with normal controls—correlating with the fact that *DPEP1* is known to be an inhibitor of cancer invasiveness in PDAC.^59^

The glutathione homeostasis is quite delicate and particularly vulnerable to any disturbances —with a decrease in intracellular glutathione concentration leading to an increased susceptibility to oxidative stress, while abundant of glutathione concentrations contribute to oxidative stress resistance, as being described in many cancer cells resulting in chemotherapy resistance.^62,63^ Indeed, in the setting of PDAC, increased concentration of glutathione was significantly associated with cell proliferation and chemotherapy resistance in cancer cells—suggesting a potential inhibitory effect on cancer growth through depletion of glutathione.^64^ Given its key role in glutathione degradation and distinctive linkages in prior studies for outcomes in PDAC, *DPEP1* is unequivocally an important gene for future research and potentially in therapeutic aspects of PDAC.

Our third hub gene, *BCAT1* plays an important role in tumorigenesis—as high expression of the Branched Chain Amino Acid Transaminase 1 (BCAT1) initiates catabolism of branched-chain amino acids (BCAAs) and generation of glutamate, leading to protein synthesis and cell growth.^65^ While expression of *BCAT1* and its isoform *BCAT2* are commonly elevated in some groups of *Kras*-related cancers (e.g., breast cancer, ovarian cancer, and lung cancer), their expression are low or even decreased in the setting of pancreatic cancer.^66,67^ The low expression of *BCAT1* and *BCAT2* in both the FPC-related PanIN groups and the SPC groups is reconfirmed in our present study, and could suggest that other genetic pathways might play a concurrent role in protein synthesis during progression of PDAC. Indeed, it was previously shown that inhibition of *BCAT1* and *BCAT2* did not impair tumor growth in PDAC, despite resulting in inhibited tumorigenesis in nonsmall cell lung cancer—indicating that tissue of origin is determinative of selective and differential pathways for BCAA and glutamine metabolism among cancer types.^66^

Although *BCAT1* seemingly cannot be used as a standalone prognostic marker in PDAC—given the well-known cellular function of BCAT1 in branched amino acid catabolism, it undoubtedly has an important regulatory role in glutamine metabolism. Due to its high connectivity with other genes, *BCAT1* may be a target gene in targeted treatment in PDAC—perhaps in combination with other mediators of BCAA and glutamine metabolism.

Finally, it is necessary to point out that, among the 76 commonly dysregulated genes detected in both FPC and SPC by the original study,^17^ only 25 overlapped with the 600 genes in the black module and 8 overlapped with the 169 genes in the gray60 module. Although with low overlapping percentage, 2 of the 3 hub genes in the gray60-based network (Fig. 4) are listed in the 76-gene panel indicating the high importance of hub genes that underlie neoplastic progression. Even though the hub genes are detectable by both single-gene-based tests and network analysis, only the latter is able to characterize their central roles for defining key molecular targets for intervention.

A limitation of the present study is the relatively low number of biologic samples comprising the gene expression profiles among both the FPC and SPC groups. Given the rareness of pancreatic samples from FPC individuals, and the low availability of PanIN-2/3 in the absence of concomitant PDAC—the inclusion of 13 FPC-related PanIN samples in the dataset seems comprehensible. The limited availability of gene expression profiles in FPC patients hindered the formation of combined gene expression datasets in the present study. Concurrently, the use of a single genome-dataset may have induced a higher comparability among gene expression profiles, thus improving overall data quality. A distinct weakness of combining different genome-dataset batches is a risk of inconsistency of data quality and comparativeness—dependent on various factors, including quality of samples and laboratory processing of samples. The coherence between present findings and results from experimental studies of PDAC cell lines may imply a validation of quality in our dataset. In addition, it is worth mentioning that the identification criteria for our hub genes were strictly based on a high amount of interconnectivity of minimum seven interconnections per gene to be considered as a hub gene.

## Conclusion

The differential expression of gene modules in FPC-related PanIN compared to SPC may implicate various aspects of cellular functions—including a decreased activity in genes related to mitochondrial structure and ATP synthesis in the FPC group, and an increased activity in extracellular structure, ECM organization, and immunologic response in the SPC group. These findings suggest that some changes in cellular activities during the formation of precursor lesions and/or PDAC formation—particularly the shift from mitochondrial ATP synthesis to glycolysis, may be more prominent in hereditary pancreatic cancers than in sporadic. Concurrently, identification of CEG modules suggests that both FPC and SPC share strong gene coexpression patterns related to intracellular activities. Identification of high-connectivity hub genes among both FPC and SPC provide basic for potential molecular targets in future treatment of hereditary and SPC. Our findings provide reference for genomic characterization of the molecular events during the development and progression of PanIN lesions to PDAC in FPC.

## Supplementary Material

Supplemental data

Supplemental data

Supplemental data

Supplemental data

Supplemental data

Supplemental data

Supplemental data

Supplemental data

## Availability of Data and Materials

All data generated or analyzed during this study are included in this published article [and its supplementary information files].

## Abbreviations Used

ATP: Adenosine triphosphate
BCAA: branched-chain amino acid
BCAT1: Branched-Chain Amino Acid Transaminase 1
CEG: commonly expressed gene
CRC: colorectal cancer
DEG: differentially expressed gene
DPEP1: Dipeptidase 1
ECM: extracellular matrix
ER: endoplasmic reticulum
FPC: familial pancreatic cancer
GEO: Gene Expression Omnibus
GO: Gene Ontology
GS: gene significance
IL22RA1: Interleukin 22 receptor, alpha 1
ME: module eigengene
MM: module membership
PanIN: pancreatic intraepithelial neoplasia
PDAC: pancreatic ductal adenocarcinoma
SPC: sporadic pancreatic cancer
TOM: topological overlap matrix
UPR: unfolded protein response
WGCNA: weighted gene coexpression network analysis
