# Supplementary figures and images for: Gene Expression Network Analysis of Precursor Lesions in Familial Pancreatic Cancer
Source: J Pancreat Cancer. 2020 Aug 5;6(1):73–84. doi: 10.1089/pancan.2020.0007 (PMC7415888; doi:10.1089/pancan.2020.0007)

# Cluster Dendrogram

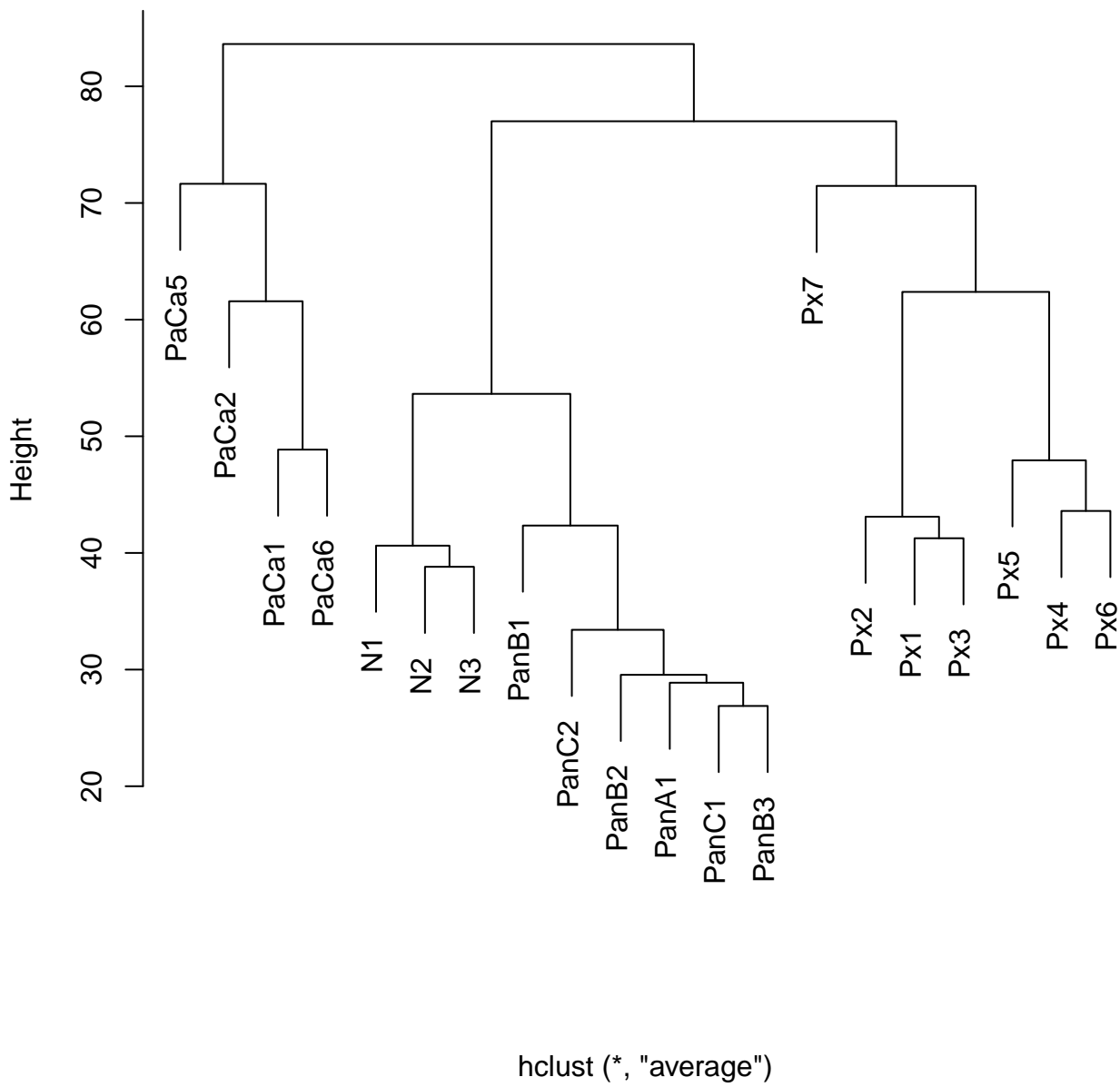

Supplement: Supplemental data [file Supp_Fig1.pdf]

### Scale independence

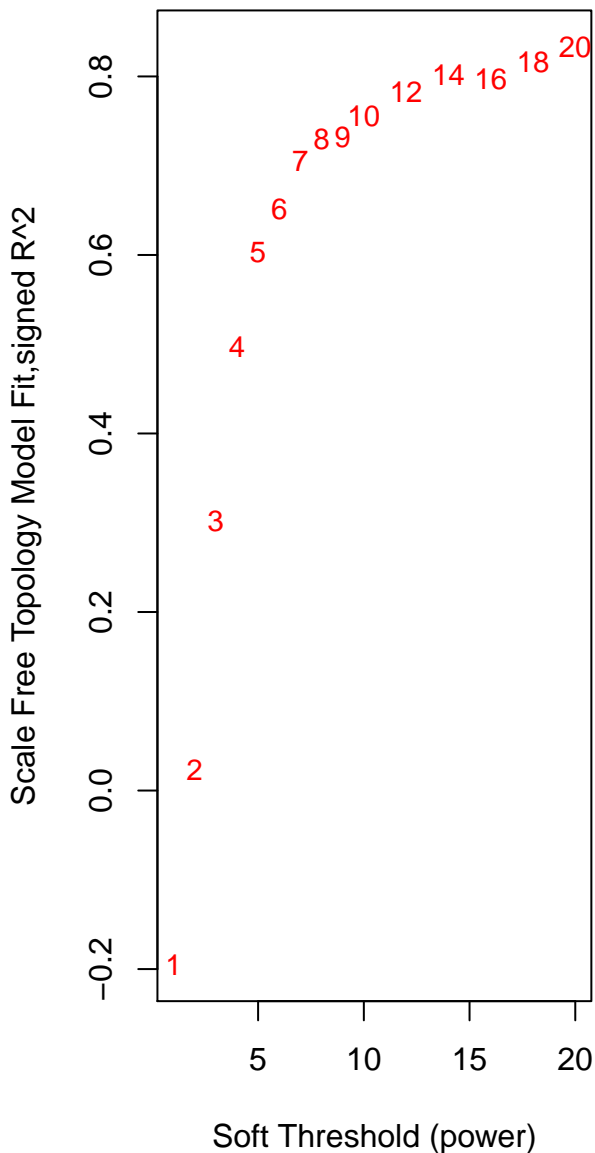

### Mean connectivity

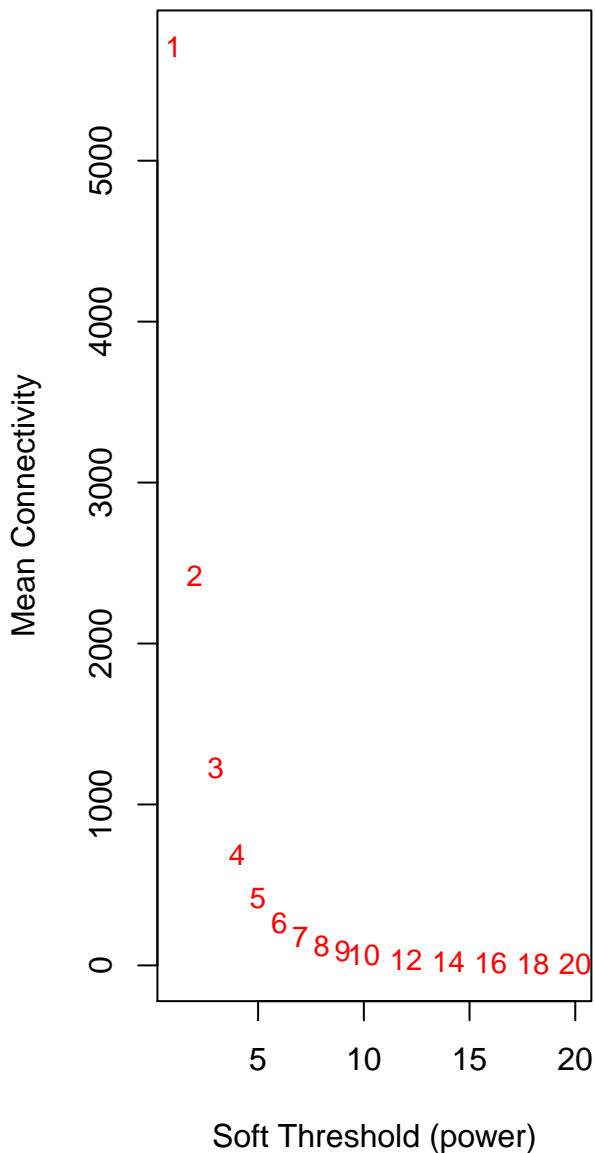

Supplement: Supplemental data [file Supp_Fig2.pdf]

# Cluster Dendrogram

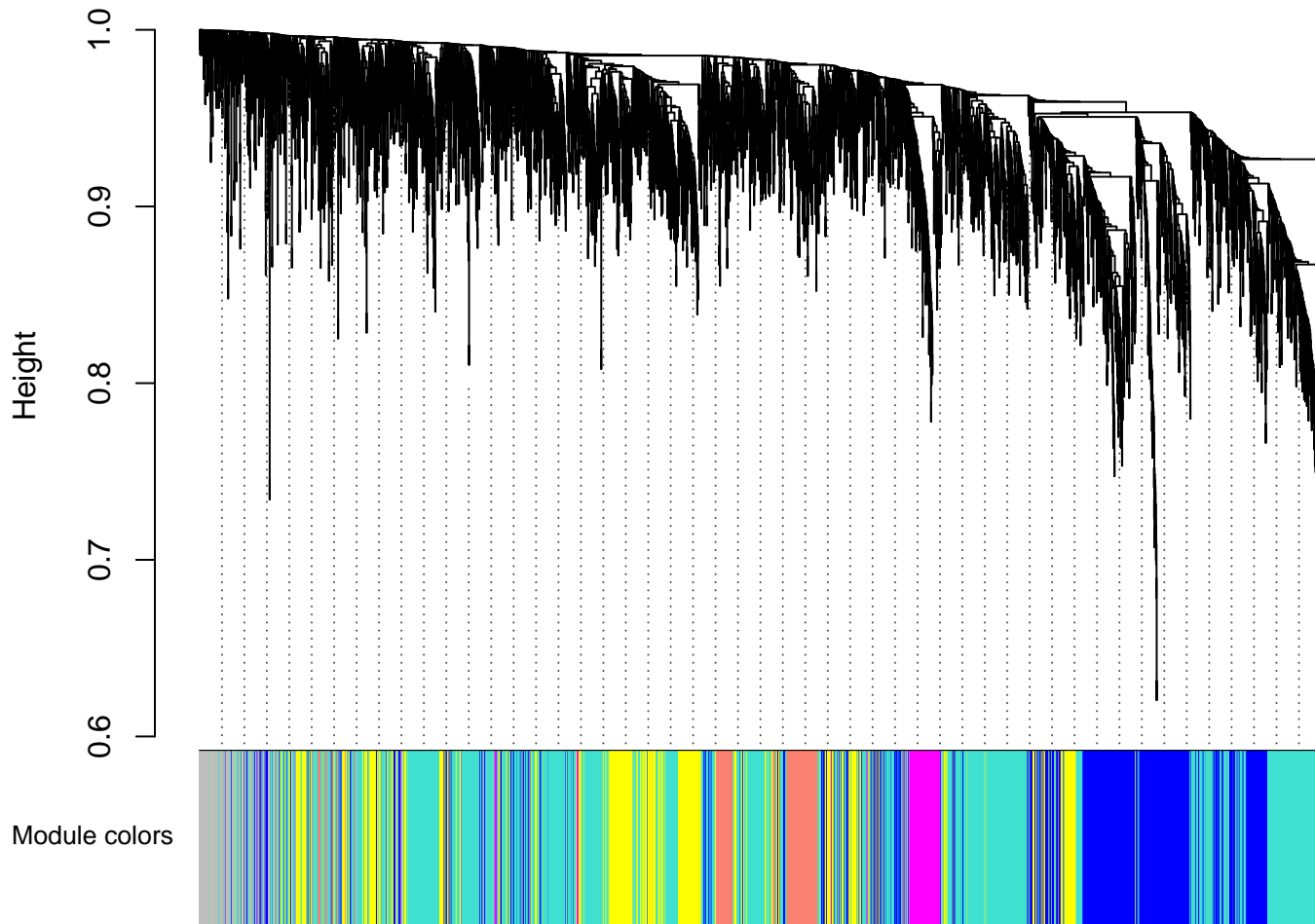

Supplement: Supplemental data [file Supp_Fig3.pdf]

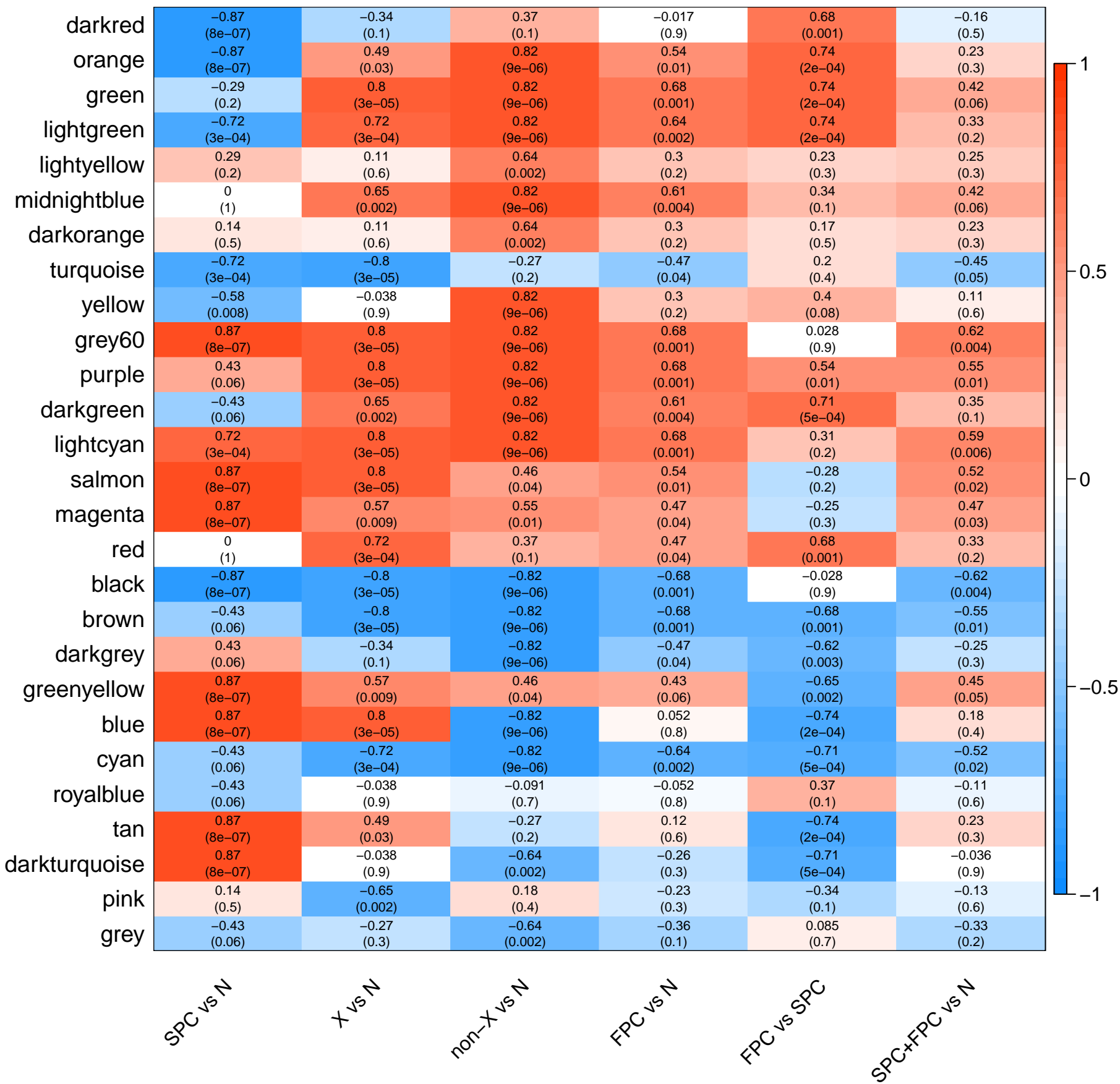

Supplement: Supplemental data [file Supp_Fig4.pdf]

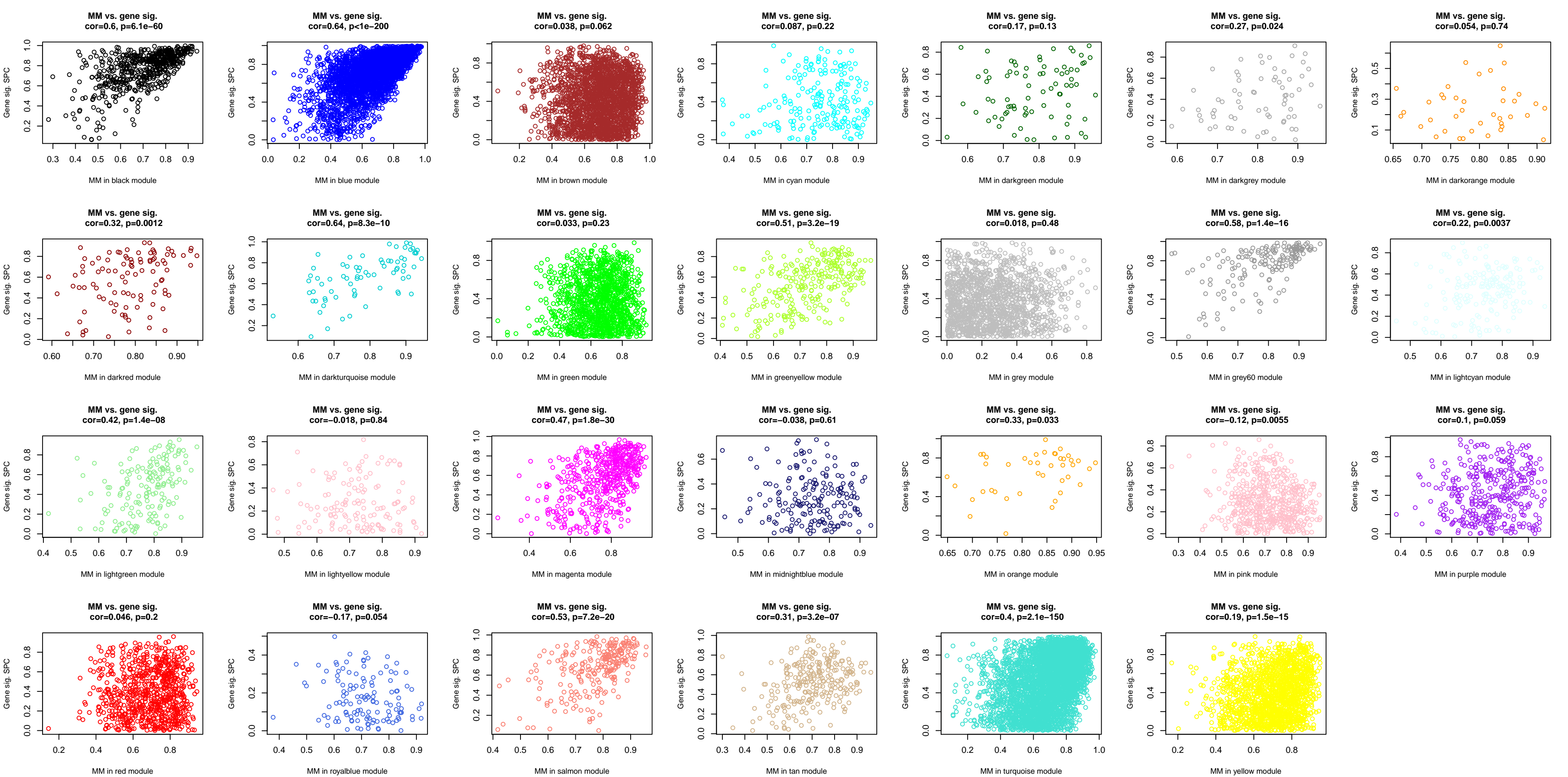

Supplement: Supplemental data [file Supp_Fig5.pdf]

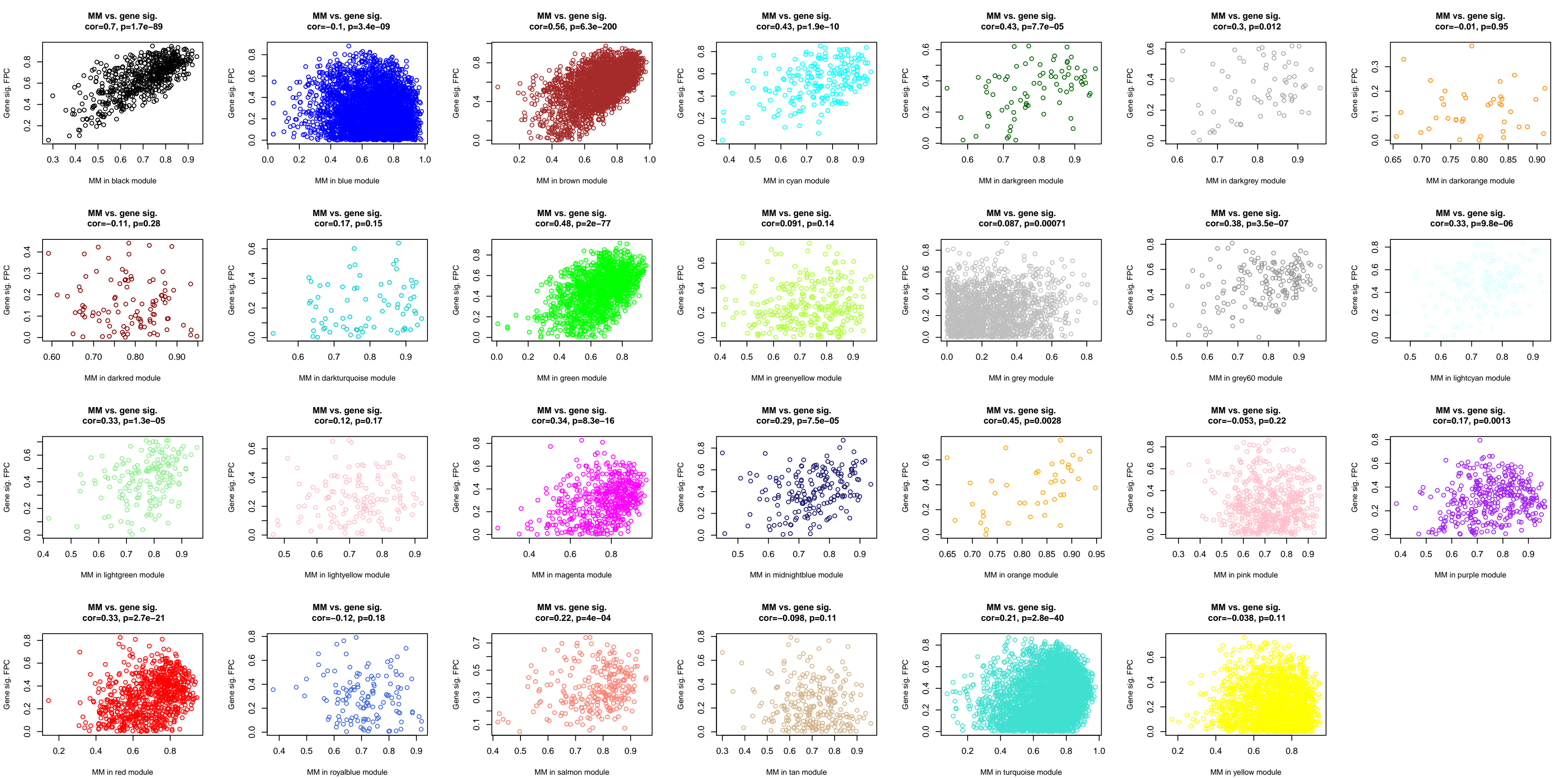

Supplement: Supplemental data [file Supp_Fig6.pdf]

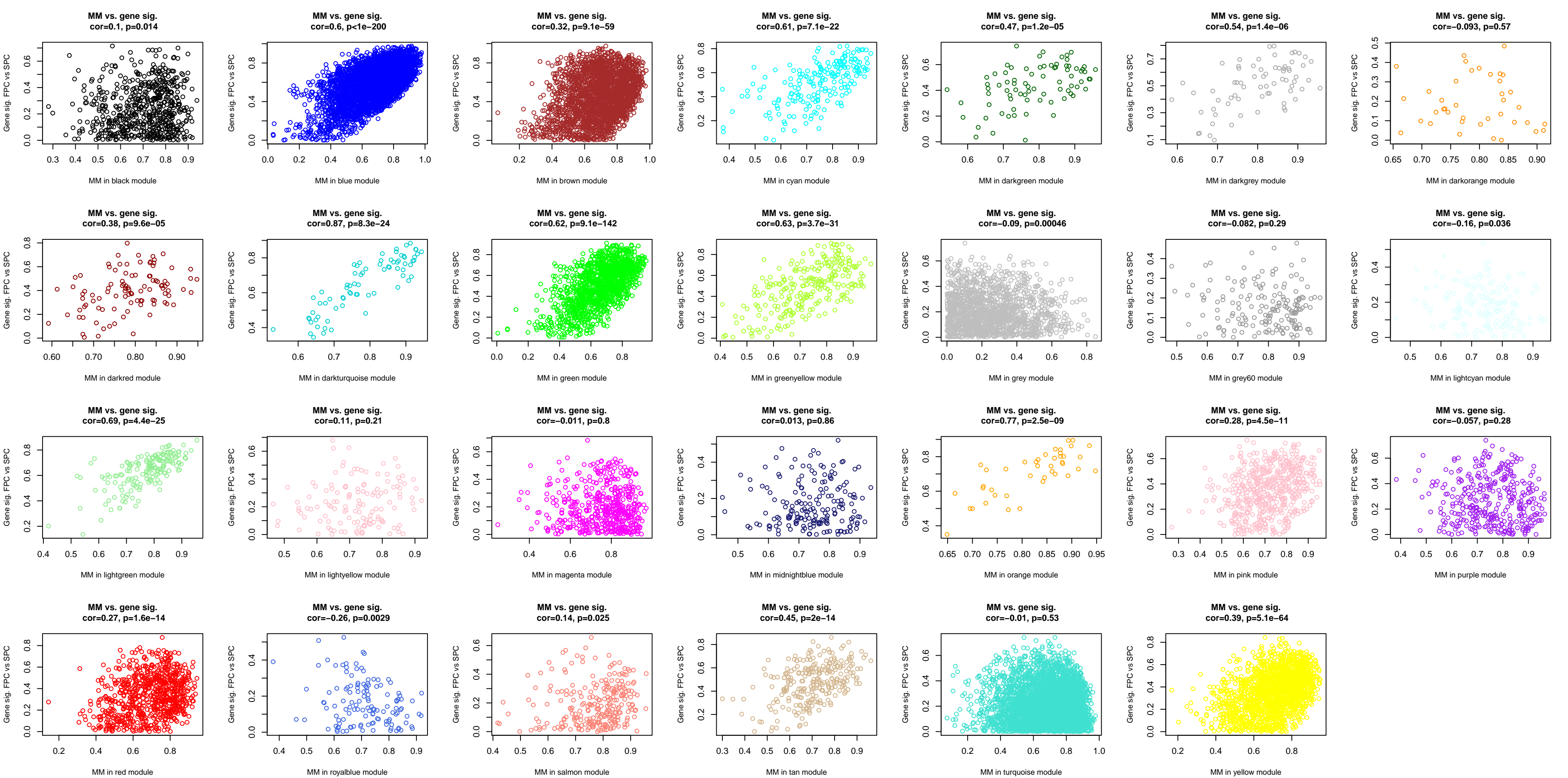

Supplement: Supplemental data [file Supp_Fig7.pdf]
