# Supplementary material for: Gene Expression Network Analysis of Precursor Lesions in Familial Pancreatic Cancer
Source: J Pancreat Cancer. 2020 Aug 5;6(1):73–84. doi: 10.1089/pancan.2020.0007 (PMC7415888; doi:10.1089/pancan.2020.0007)
Supplement: Supplemental data [file Supp_Table1.pdf]

| module | modSize | bkgrModSize | rank | enrichmer | Bonferoni | nModGe | bkgrTerr | termID | termName |
|---|---|---|---|---|---|---|---|---|---|
| black | 599 | 511 | 1 | 4.41E-12 | 7.56E-08 | 19 | 79 | GO:0006613 | cotranslational protein targeting to membrane |
| black | 599 | 511 | 2 | 9.00E-12 | 1.54E-07 | 19 | 82 | GO:0045047 | protein targeting to ER |
| black | 599 | 511 | 3 | 1.32E-11 | 2.26E-07 | 18 | 74 | GO:0006614 | SRP-dependent cotranslational protein targeting to membrane |
| black | 599 | 511 | 4 | 2.21E-11 | 3.79E-07 | 19 | 86 | GO:0072599 | establishment of protein localization to endoplasmic reticulum |
| black | 599 | 511 | 5 | 1.97E-10 | 3.37E-06 | 20 | 108 | GO:0070972 | protein localization to endoplasmic reticulum |
| black | 599 | 511 | 6 | 1.40E-09 | 2.39E-05 | 51 | 634 | GO:0043604 | amide biosynthetic process |
| black | 599 | 511 | 7 | 1.46E-09 | 2.50E-05 | 54 | 694 | GO:0006518 | peptide metabolic process |
| black | 599 | 511 | 8 | 1.54E-09 | 2.63E-05 | 457 | 12690 | GO:0005622 | intracellular |
| black | 599 | 511 | 9 | 1.97E-09 | 3.37E-05 | 21 | 135 | GO:0003735 | structural constituent of ribosome |
| black | 599 | 511 | 10 | 2.21E-09 | 3.78E-05 | 46 | 546 | GO:0006412 | translation |
| blue | 3472 | 3025 | 1 | 4.41E-16 | 7.56E-12 | 117 | 301 | GO:0043062 | extracellular structure organization |
| blue | 3472 | 3025 | 2 | 9.27E-16 | 1.59E-11 | 116 | 300 | GO:0030198 | extracellular matrix organization |
| blue | 3472 | 3025 | 3 | 7.77E-15 | 1.33E-10 | 136 | 382 | GO:0030055 | cell-substrate junction |
| blue | 3472 | 3025 | 4 | 1.21E-14 | 2.07E-10 | 899 | 3865 | GO:0031982 | vesicle |
| blue | 3472 | 3025 | 5 | 1.71E-14 | 2.93E-10 | 133 | 374 | GO:0005925 | focal adhesion |
| blue | 3472 | 3025 | 6 | 3.45E-14 | 5.91E-10 | 133 | 377 | GO:0005924 | cell-substrate adherens junction |
| blue | 3472 | 3025 | 7 | 1.14E-13 | 1.95E-09 | 38 | 61 | GO:0005518 | collagen binding |
| blue | 3472 | 3025 | 8 | 4.64E-13 | 7.95E-09 | 155 | 475 | GO:0031012 | extracellular matrix |
| blue | 3472 | 3025 | 9 | 6.78E-13 | 1.16E-08 | 154 | 473 | GO:0005912 | adherens junction |
| blue | 3472 | 3025 | 10 | 3.03E-12 | 5.19E-08 | 609 | 2533 | GO:1903561 | extracellular vesicle |
| brown | 2419 | 1876 | 1 | 7.45E-05 | 1 | 102 | 600 | GO:0043547 | positive regulation of GTPase activity |
| brown | 2419 | 1876 | 2 | 0.00015 | 1 | 49 | 246 | GO:0005096 | GTPase activator activity |
| brown | 2419 | 1876 | 3 | 0.00022 | 1 | 107 | 653 | GO:0043087 | regulation of GTPase activity |
| brown | 2419 | 1876 | 4 | 0.00035 | 1 | 52 | 275 | GO:0030695 | GTPase regulator activity |
| brown | 2419 | 1876 | 5 | 0.00088 | 1 | 10 | 28 | GO:0043368 | positive T cell selection |
| brown | 2419 | 1876 | 6 | 0.00093 | 1 | 5 | 8 | GO:1903800 | positive regulation of production of miRNAs involved in gene silencing by miRNA |
| brown | 2419 | 1876 | 7 | 0.00163 | 1 | 3 | 3 | GO:0018916 | nitrobenzene metabolic process |
| brown | 2419 | 1876 | 8 | 0.00163 | 1 | 3 | 3 | GO:0046604 | positive regulation of mitotic centrosome separation |
| brown | 2419 | 1876 | 9 | 0.00163 | 1 | 3 | 3 | GO:0051410 | detoxification of nitrogen compound |
| brown | 2419 | 1876 | 10 | 0.00163 | 1 | 3 | 3 | GO:0061205 | paramesonephric duct development |
| cyan | 201 | 166 | 1 | 2.60E-16 | 4.44E-12 | 30 | 445 | GO:0005743 | mitochondrial inner membrane |
| cyan | 201 | 166 | 2 | 6.00E-15 | 1.03E-10 | 30 | 500 | GO:0019866 | organelle inner membrane |

| | | | | | | | | | |
|---|---|---|---|---|---|---|---|---|---|
| cyan | 201 | 166 | 3 | 4.51E-14 | 7.73E-10 | 33 | 662 | GO:0005740 | mitochondrial envelope |
| cyan | 201 | 166 | 4 | 5.84E-14 | 9.99E-10 | 32 | 626 | GO:0031966 | mitochondrial membrane |
| cyan | 201 | 166 | 5 | 9.23E-14 | 1.58E-09 | 20 | 215 | GO:0046034 | ATP metabolic process |
| cyan | 201 | 166 | 6 | 1.46E-13 | 2.50E-09 | 50 | 1553 | GO:0005739 | mitochondrion |
| cyan | 201 | 166 | 7 | 1.10E-12 | 1.88E-08 | 13 | 77 | GO:0042775 | mitochondrial ATP synthesis coupled electron transport |
| cyan | 201 | 166 | 8 | 1.17E-12 | 2.00E-08 | 14 | 96 | GO:0006119 | oxidative phosphorylation |
| cyan | 201 | 166 | 9 | 4.70E-12 | 8.04E-08 | 8 | 18 | GO:0042776 | mitochondrial ATP synthesis coupled proton transport |
| cyan | 201 | 166 | 10 | 6.49E-12 | 1.11E-07 | 13 | 88 | GO:1902600 | hydrogen ion transmembrane transport |
| darkgree | 79 | 74 | 1 | 0.00027 | 1 | 7 | 272 | GO:0008202 | steroid metabolic process |
| darkgree | 79 | 74 | 2 | 0.00052 | 1 | 4 | 80 | GO:0004497 | monooxygenase activity |
| darkgree | 79 | 74 | 3 | 0.00059 | 1 | 2 | 8 | GO:0052695 | cellular glucuronidation |
| darkgree | 79 | 74 | 4 | 0.00127 | 1 | 3 | 46 | GO:0016836 | hydro-lyase activity |
| darkgree | 79 | 74 | 5 | 0.00136 | 1 | 2 | 12 | GO:0031996 | thioesterase binding |
| darkgree | 79 | 74 | 6 | 0.00143 | 1 | 31 | 4053 | GO:0005576 | extracellular region |
| darkgree | 79 | 74 | 7 | 0.00245 | 1 | 2 | 16 | GO:0008392 | arachidonic acid epoxygenase activity |
| darkgree | 79 | 74 | 8 | 0.00262 | 1 | 8 | 515 | GO:0032787 | monocarboxylic acid metabolic process |
| darkgree | 79 | 74 | 9 | 0.00357 | 1 | 3 | 66 | GO:0019730 | antimicrobial humoral response |
| darkgree | 79 | 74 | 10 | 0.00383 | 1 | 2 | 20 | GO:0019373 | epoxygenase P450 pathway |
| darkgrey | 70 | 62 | 1 | 5.68E-05 | 0.97321 | 4 | 54 | GO:0060291 | long-term synaptic potentiation |
| darkgrey | 70 | 62 | 2 | 0.00022 | 1 | 2 | 6 | GO:0033211 | adiponectin-activated signaling pathway |
| darkgrey | 70 | 62 | 3 | 0.00032 | 1 | 4 | 84 | GO:0005913 | cell-cell adherens junction |
| darkgrey | 70 | 62 | 4 | 0.00035 | 1 | 15 | 1453 | GO:0007267 | cell-cell signaling |
| darkgrey | 70 | 62 | 5 | 0.00066 | 1 | 2 | 10 | GO:0070254 | mucus secretion |
| darkgrey | 70 | 62 | 6 | 0.00071 | 1 | 10 | 769 | GO:0010243 | response to organonitrogen compound |
| darkgrey | 70 | 62 | 7 | 0.00077 | 1 | 4 | 106 | GO:0043200 | response to amino acid |
| darkgrey | 70 | 62 | 8 | 0.00082 | 1 | 13 | 1240 | GO:0007155 | cell adhesion |
| darkgrey | 70 | 62 | 9 | 0.00096 | 1 | 2 | 12 | GO:0006122 | mitochondrial electron transport, ubiquinol to cytochrome c |
| darkgrey | 70 | 62 | 10 | 0.00111 | 1 | 4 | 117 | GO:0006633 | fatty acid biosynthetic process |
| darkorar | 39 | 28 | 1 | 0.00024 | 1 | 3 | 69 | GO:0005905 | clathrin-coated pit |
| darkorar | 39 | 28 | 2 | 0.00102 | 1 | 2 | 27 | GO:0031904 | endosome lumen |
| darkorar | 39 | 28 | 3 | 0.00176 | 1 | 1 | 1 | GO:0033883 | pyridoxal phosphatase activity |
| darkorar | 39 | 28 | 4 | 0.00176 | 1 | 1 | 1 | GO:0046570 | methylthioribulose 1-phosphate dehydratase activity |
| darkorar | 39 | 28 | 5 | 0.00269 | 1 | 2 | 44 | GO:0036297 | interstrand cross-link repair |

| | | | | | | | | | |
|---|---|---|---|---|---|---|---|---|---|
| darkorai | 39 | 28 | 6 | 0.00333 | 1 | 2 | 49 | GO:0004402 | histone acetyltransferase activity |
| darkorai | 39 | 28 | 7 | 0.00346 | 1 | 2 | 50 | GO:0061733 | peptide-lysine-N-acetyltransferase activity |
| darkorai | 39 | 28 | 8 | 0.00351 | 1 | 1 | 2 | GO:0004174 | electron-transferring-flavoprotein dehydrogenase activity |
| darkorai | 39 | 28 | 9 | 0.00351 | 1 | 1 | 2 | GO:0042328 | heparan sulfate N-acetylglucosaminyltransferase activity |
| darkorai | 39 | 28 | 10 | 0.00351 | 1 | 1 | 2 | GO:0050509 | N-acetylglucosaminyl-proteoglycan 4-beta-glucuronosyltransferase activity |
| darkred | 100 | 85 | 1 | 2.81E-05 | 0.48166 | 2 | 2 | GO:0030283 | testosterone dehydrogenase [NAD(P)] activity |
| darkred | 100 | 85 | 2 | 8.41E-05 | 1 | 2 | 3 | GO:0018963 | phthalate metabolic process |
| darkred | 100 | 85 | 3 | 0.00017 | 1 | 2 | 4 | GO:0018879 | biphenyl metabolic process |
| darkred | 100 | 85 | 4 | 0.00017 | 1 | 2 | 4 | GO:0018894 | dibenzo-p-dioxin metabolic process |
| darkred | 100 | 85 | 5 | 0.00042 | 1 | 2 | 6 | GO:0061370 | testosterone biosynthetic process |
| darkred | 100 | 85 | 6 | 0.00059 | 1 | 16 | 1210 | GO:1902494 | catalytic complex |
| darkred | 100 | 85 | 7 | 0.00072 | 1 | 4 | 76 | GO:0008080 | N-acetyltransferase activity |
| darkred | 100 | 85 | 8 | 0.00084 | 1 | 4 | 79 | GO:0000123 | histone acetyltransferase complex |
| darkred | 100 | 85 | 9 | 0.00125 | 1 | 4 | 88 | GO:0016410 | N-acyltransferase activity |
| darkred | 100 | 85 | 10 | 0.00148 | 1 | 4 | 92 | GO:0016407 | acetyltransferase activity |
| darkturc | 74 | 50 | 1 | 3.09E-11 | 5.30E-07 | 5 | 9 | GO:0042571 | immunoglobulin complex, circulating |
| darkturc | 74 | 50 | 2 | 1.93E-10 | 3.31E-06 | 5 | 12 | GO:0034987 | immunoglobulin receptor binding |
| darkturc | 74 | 50 | 3 | 6.05E-08 | 0.00104 | 6 | 67 | GO:0003823 | antigen binding |
| darkturc | 74 | 50 | 4 | 1.32E-07 | 0.00226 | 5 | 39 | GO:0006958 | complement activation, classical pathway |
| darkturc | 74 | 50 | 5 | 3.44E-07 | 0.0059 | 5 | 47 | GO:0002455 | humoral immune response mediated by circulating immunoglobulin |
| darkturc | 74 | 50 | 6 | 4.94E-07 | 0.00846 | 4 | 21 | GO:0006910 | phagocytosis, recognition |
| darkturc | 74 | 50 | 7 | 1.19E-06 | 0.02036 | 5 | 60 | GO:0006956 | complement activation |
| darkturc | 74 | 50 | 8 | 1.73E-06 | 0.02956 | 7 | 186 | GO:0006959 | humoral immune response |
| darkturc | 74 | 50 | 9 | 5.25E-06 | 0.08993 | 4 | 37 | GO:0006911 | phagocytosis, engulfment |
| darkturc | 74 | 50 | 10 | 9.65E-06 | 0.16528 | 2 | 2 | GO:0071756 | pentameric IgM immunoglobulin complex |
| green | 1325 | 1065 | 1 | 2.61E-07 | 0.00446 | 121 | 1149 | GO:0004872 | receptor activity |
| green | 1325 | 1065 | 2 | 6.53E-07 | 0.01118 | 25 | 125 | GO:0015276 | ligand-gated ion channel activity |
| green | 1325 | 1065 | 3 | 1.09E-06 | 0.01873 | 90 | 810 | GO:0004888 | transmembrane signaling receptor activity |
| green | 1325 | 1065 | 4 | 4.13E-06 | 0.07067 | 22 | 112 | GO:0009593 | detection of chemical stimulus |
| green | 1325 | 1065 | 5 | 6.03E-06 | 0.10331 | 18 | 82 | GO:0050907 | detection of chemical stimulus involved in sensory perception |
| green | 1325 | 1065 | 6 | 9.12E-06 | 0.15605 | 23 | 126 | GO:0007606 | sensory perception of chemical stimulus |
| green | 1325 | 1065 | 7 | 9.12E-06 | 0.15605 | 23 | 126 | GO:0050906 | detection of stimulus involved in sensory perception |
| green | 1325 | 1065 | 8 | 1.62E-05 | 0.27801 | 94 | 914 | GO:0038023 | signaling receptor activity |

| | | | | | | | | | |
|---|---|---|---|---|---|---|---|---|---|
| green | 1325 | 1065 | 9 | 2.09E-05 | 0.35699 | 37 | 268 | GO:0051606 | detection of stimulus |
| green | 1325 | 1065 | 10 | 3.40E-05 | 0.58284 | 16 | 76 | GO:0030594 | neurotransmitter receptor activity |
| greenye | 268 | 242 | 1 | 1.81E-05 | 0.30979 | 6 | 37 | GO:0042304 | regulation of fatty acid biosynthetic process |
| greenye | 268 | 242 | 2 | 2.51E-05 | 0.42943 | 50 | 1831 | GO:0008283 | cell proliferation |
| greenye | 268 | 242 | 3 | 7.38E-05 | 1 | 14 | 272 | GO:0008202 | steroid metabolic process |
| greenye | 268 | 242 | 4 | 8.31E-05 | 1 | 6 | 48 | GO:0006695 | cholesterol biosynthetic process |
| greenye | 268 | 242 | 5 | 8.34E-05 | 1 | 17 | 384 | GO:0005911 | cell-cell junction |
| greenye | 268 | 242 | 6 | 9.09E-05 | 1 | 7 | 70 | GO:0000786 | nucleosome |
| greenye | 268 | 242 | 7 | 9.79E-05 | 1 | 88 | 4053 | GO:0005576 | extracellular region |
| greenye | 268 | 242 | 8 | 0.00013 | 1 | 40 | 1439 | GO:0042127 | regulation of cell proliferation |
| greenye | 268 | 242 | 9 | 0.00013 | 1 | 5 | 33 | GO:0019731 | antibacterial humoral response |
| greenye | 268 | 242 | 10 | 0.00014 | 1 | 10 | 156 | GO:0006694 | steroid biosynthetic process |
| grey | 1512 | 1214 | 1 | 0.00021 | 1 | 1014 | 12690 | GO:0005622 | intracellular |
| grey | 1512 | 1214 | 2 | 0.00031 | 1 | 31 | 211 | GO:0000075 | cell cycle checkpoint |
| grey | 1512 | 1214 | 3 | 0.00033 | 1 | 7 | 19 | GO:0070987 | error-free translesion synthesis |
| grey | 1512 | 1214 | 4 | 0.00034 | 1 | 53 | 430 | GO:0007067 | mitotic nuclear division |
| grey | 1512 | 1214 | 5 | 0.00071 | 1 | 92 | 868 | GO:1903047 | mitotic cell cycle process |
| grey | 1512 | 1214 | 6 | 0.00072 | 1 | 73 | 658 | GO:0070925 | organelle assembly |
| grey | 1512 | 1214 | 7 | 0.00074 | 1 | 99 | 948 | GO:0000278 | mitotic cell cycle |
| grey | 1512 | 1214 | 8 | 0.00079 | 1 | 20 | 121 | GO:0007062 | sister chromatid cohesion |
| grey | 1512 | 1214 | 9 | 0.00084 | 1 | 759 | 9276 | GO:0044237 | cellular metabolic process |
| grey | 1512 | 1214 | 10 | 0.00098 | 1 | 109 | 1070 | GO:0051276 | chromosome organization |
| grey60 | 169 | 155 | 1 | 3.59E-06 | 0.06142 | 3 | 4 | GO:0071953 | elastic fiber |
| grey60 | 169 | 155 | 2 | 1.89E-05 | 0.32403 | 31 | 1439 | GO:0042127 | regulation of cell proliferation |
| grey60 | 169 | 155 | 3 | 2.95E-05 | 0.50477 | 14 | 392 | GO:0006979 | response to oxidative stress |
| grey60 | 169 | 155 | 4 | 0.0001 | 1 | 3 | 10 | GO:0070102 | interleukin-6-mediated signaling pathway |
| grey60 | 169 | 155 | 5 | 0.00011 | 1 | 4 | 26 | GO:0071354 | cellular response to interleukin-6 |
| grey60 | 169 | 155 | 6 | 0.00014 | 1 | 34 | 1831 | GO:0008283 | cell proliferation |
| grey60 | 169 | 155 | 7 | 0.00019 | 1 | 3 | 12 | GO:0006264 | mitochondrial DNA replication |
| grey60 | 169 | 155 | 8 | 0.00019 | 1 | 4 | 30 | GO:0070741 | response to interleukin-6 |
| grey60 | 169 | 155 | 9 | 0.00025 | 1 | 4 | 32 | GO:0044091 | membrane biogenesis |
| grey60 | 169 | 155 | 10 | 0.00028 | 1 | 2 | 3 | GO:0090298 | negative regulation of mitochondrial DNA replication |
| lightcyar | 172 | 125 | 1 | 2.37E-05 | 0.40545 | 4 | 22 | GO:0048679 | regulation of axon regeneration |

| | | | | | | | | | |
|---|---|---|---|---|---|---|---|---|---|
| lightcyan | 172 | 125 | 2 | 4.02E-05 | 0.68849 | 4 | 25 | GO:0070570 | regulation of neuron projection regeneration |
| lightcyan | 172 | 125 | 3 | 9.85E-05 | 1 | 3 | 12 | GO:0048681 | negative regulation of axon regeneration |
| lightcyan | 172 | 125 | 4 | 0.00013 | 1 | 3 | 13 | GO:0070571 | negative regulation of neuron projection regeneration |
| lightcyan | 172 | 125 | 5 | 0.00027 | 1 | 4 | 40 | GO:0031103 | axon regeneration |
| lightcyan | 172 | 125 | 6 | 0.00032 | 1 | 5 | 76 | GO:0007098 | centrosome cycle |
| lightcyan | 172 | 125 | 7 | 0.00054 | 1 | 4 | 48 | GO:0031102 | neuron projection regeneration |
| lightcyan | 172 | 125 | 8 | 0.0009 | 1 | 2 | 6 | GO:0002318 | myeloid progenitor cell differentiation |
| lightcyan | 172 | 125 | 9 | 0.00125 | 1 | 2 | 7 | GO:0035374 | chondroitin sulfate binding |
| lightcyan | 172 | 125 | 10 | 0.00133 | 1 | 4 | 61 | GO:0048678 | response to axon injury |
| lightgree | 168 | 150 | 1 | 8.20E-06 | 0.14047 | 11 | 222 | GO:0060485 | mesenchyme development |
| lightgree | 168 | 150 | 2 | 1.60E-05 | 0.2744 | 3 | 6 | GO:0019237 | centromeric DNA binding |
| lightgree | 168 | 150 | 3 | 0.0007 | 1 | 5 | 75 | GO:0061333 | renal tubule morphogenesis |
| lightgree | 168 | 150 | 4 | 0.00098 | 1 | 4 | 47 | GO:0009948 | anterior/posterior axis specification |
| lightgree | 168 | 150 | 5 | 0.00124 | 1 | 4 | 50 | GO:0005876 | spindle microtubule |
| lightgree | 168 | 150 | 6 | 0.00176 | 1 | 5 | 92 | GO:0061326 | renal tubule development |
| lightgree | 168 | 150 | 7 | 0.00179 | 1 | 2 | 7 | GO:0014866 | skeletal myofibril assembly |
| lightgree | 168 | 150 | 8 | 0.00179 | 1 | 2 | 7 | GO:0003696 | satellite DNA binding |
| lightgree | 168 | 150 | 9 | 0.00213 | 1 | 5 | 96 | GO:0001657 | ureteric bud development |
| lightgree | 168 | 150 | 10 | 0.00213 | 1 | 5 | 96 | GO:0072163 | mesonephric epithelium development |
| lightyell | 133 | 104 | 1 | 0.00025 | 1 | 2 | 4 | GO:0061304 | retinal blood vessel morphogenesis |
| lightyell | 133 | 104 | 2 | 0.00042 | 1 | 2 | 5 | GO:0035426 | extracellular matrix-cell signaling |
| lightyell | 133 | 104 | 3 | 0.00147 | 1 | 2 | 9 | GO:0061299 | retina vasculature morphogenesis in camera-type eye |
| lightyell | 133 | 104 | 4 | 0.00184 | 1 | 2 | 10 | GO:0031987 | locomotion involved in locomotory behavior |
| lightyell | 133 | 104 | 5 | 0.00223 | 1 | 2 | 11 | GO:0005513 | detection of calcium ion |
| lightyell | 133 | 104 | 6 | 0.00267 | 1 | 2 | 12 | GO:0019215 | intermediate filament binding |
| lightyell | 133 | 104 | 7 | 0.00365 | 1 | 2 | 14 | GO:0042813 | Wnt-activated receptor activity |
| lightyell | 133 | 104 | 8 | 0.0041 | 1 | 9 | 479 | GO:0007423 | sensory organ development |
| lightyell | 133 | 104 | 9 | 0.00419 | 1 | 2 | 15 | GO:0001673 | male germ cell nucleus |
| lightyell | 133 | 104 | 10 | 0.00477 | 1 | 2 | 16 | GO:0061298 | retina vasculature development in camera-type eye |
| magenta | 530 | 478 | 1 | 1.86E-15 | 3.19E-11 | 144 | 2533 | GO:1903561 | extracellular vesicle |
| magenta | 530 | 478 | 2 | 6.74E-15 | 1.15E-10 | 142 | 2520 | GO:0070062 | extracellular exosome |
| magenta | 530 | 478 | 3 | 1.89E-13 | 3.24E-09 | 187 | 3865 | GO:0031982 | vesicle |
| magenta | 530 | 478 | 4 | 1.55E-08 | 0.00027 | 169 | 3846 | GO:0012505 | endomembrane system |

| | | | | | | | | | |
|---|---|---|---|---|---|---|---|---|---|
| magenta | 530 | 478 | 5 | 1.39E-07 | 0.00238 | 172 | 4053 | GO:0005576 | extracellular region |
| magenta | 530 | 478 | 6 | 1.93E-07 | 0.00331 | 32 | 384 | GO:0005911 | cell-cell junction |
| magenta | 530 | 478 | 7 | 1.43E-06 | 0.02445 | 283 | 7739 | GO:0016020 | membrane |
| magenta | 530 | 478 | 8 | 4.10E-06 | 0.07012 | 73 | 1428 | GO:0046903 | secretion |
| magenta | 530 | 478 | 9 | 4.65E-06 | 0.07969 | 7 | 24 | GO:0032528 | microvillus organization |
| magenta | 530 | 478 | 10 | 6.70E-06 | 0.11473 | 31 | 431 | GO:0050839 | cell adhesion molecule binding |
| midnigh | 181 | 122 | 1 | 0.00017 | 1 | 2 | 3 | GO:0005157 | macrophage colony-stimulating factor receptor binding |
| midnigh | 181 | 122 | 2 | 0.00429 | 1 | 2 | 13 | GO:0001741 | XY body |
| midnigh | 181 | 122 | 3 | 0.00734 | 1 | 2 | 17 | GO:0007026 | negative regulation of microtubule depolymerization |
| midnigh | 181 | 122 | 4 | 0.00734 | 1 | 2 | 17 | GO:0009264 | deoxyribonucleotide catabolic process |
| midnigh | 181 | 122 | 5 | 0.00766 | 1 | 1 | 1 | GO:0016077 | snoRNA catabolic process |
| midnigh | 181 | 122 | 6 | 0.00766 | 1 | 1 | 1 | GO:0033499 | galactose catabolic process via UDP-galactose |
| midnigh | 181 | 122 | 7 | 0.00766 | 1 | 1 | 1 | GO:0035863 | dITP catabolic process |
| midnigh | 181 | 122 | 8 | 0.00766 | 1 | 1 | 1 | GO:0055113 | epiboly involved in gastrulation with mouth forming second |
| midnigh | 181 | 122 | 9 | 0.00766 | 1 | 1 | 1 | GO:0070143 | mitochondrial alanyl-tRNA aminoacylation |
| midnigh | 181 | 122 | 10 | 0.00766 | 1 | 1 | 1 | GO:1901639 | XDP catabolic process |
| orange | 41 | 33 | 1 | 0.00085 | 1 | 2 | 21 | GO:0046697 | decidualization |
| orange | 41 | 33 | 2 | 0.00199 | 1 | 2 | 32 | GO:0001893 | maternal placenta development |
| orange | 41 | 33 | 3 | 0.00207 | 1 | 1 | 1 | GO:0048685 | negative regulation of collateral sprouting of intact axon in response to injury |
| orange | 41 | 33 | 4 | 0.00207 | 1 | 1 | 1 | GO:1902773 | GTPase activator complex |
| orange | 41 | 33 | 5 | 0.00207 | 1 | 1 | 1 | GO:0001631 | cysteinyl leukotriene receptor activity |
| orange | 41 | 33 | 6 | 0.00207 | 1 | 1 | 1 | GO:0004051 | arachidonate 5-lipoxygenase activity |
| orange | 41 | 33 | 7 | 0.00207 | 1 | 1 | 1 | GO:0031682 | G-protein gamma-subunit binding |
| orange | 41 | 33 | 8 | 0.00207 | 1 | 1 | 1 | GO:0047395 | glycerophosphoinositol glycerophosphodiesterase activity |
| orange | 41 | 33 | 9 | 0.00294 | 1 | 2 | 39 | GO:0043551 | regulation of phosphatidylinositol 3-kinase activity |
| orange | 41 | 33 | 10 | 0.0034 | 1 | 2 | 42 | GO:0007566 | embryo implantation |
| pink | 534 | 366 | 1 | 0.00053 | 1 | 2 | 2 | GO:0036228 | protein targeting to nuclear inner membrane |
| pink | 534 | 366 | 2 | 0.00156 | 1 | 2 | 3 | GO:0033693 | neurofilament bundle assembly |
| pink | 534 | 366 | 3 | 0.00156 | 1 | 2 | 3 | GO:0016035 | zeta DNA polymerase complex |
| pink | 534 | 366 | 4 | 0.00173 | 1 | 3 | 11 | GO:0000800 | lateral element |
| pink | 534 | 366 | 5 | 0.00258 | 1 | 7 | 81 | GO:0000794 | condensed nuclear chromosome |
| pink | 534 | 366 | 6 | 0.0029 | 1 | 3 | 13 | GO:0003906 | DNA-(apurinic or apyrimidinic site) lyase activity |
| pink | 534 | 366 | 7 | 0.00306 | 1 | 2 | 4 | GO:1902953 | positive regulation of ER to Golgi vesicle-mediated transport |

| | | | | | | | | | |
|---|---|---|---|---|---|---|---|---|---|
| pink | 534 | 366 | 8 | 0.00363 | 1 | 3 | 14 | GO:0055106 | ubiquitin-protein transferase regulator activity |
| pink | 534 | 366 | 9 | 0.00503 | 1 | 2 | 5 | GO:0097418 | neurofibrillary tangle |
| pink | 534 | 366 | 10 | 0.00663 | 1 | 4 | 33 | GO:0043928 | exonucleolytic nuclear-transcribed mRNA catabolic process in deadenylation-dependent decay |
| purple | 356 | 314 | 1 | 1.19E-06 | 0.02039 | 33 | 668 | GO:0009986 | cell surface |
| purple | 356 | 314 | 2 | 1.30E-06 | 0.02231 | 42 | 971 | GO:0048878 | chemical homeostasis |
| purple | 356 | 314 | 3 | 3.96E-06 | 0.06779 | 8 | 48 | GO:0033762 | response to glucagon |
| purple | 356 | 314 | 4 | 8.59E-06 | 0.14713 | 36 | 835 | GO:0033993 | response to lipid |
| purple | 356 | 314 | 5 | 2.23E-05 | 0.38115 | 180 | 7293 | GO:0050896 | response to stimulus |
| purple | 356 | 314 | 6 | 2.40E-05 | 0.41041 | 118 | 4304 | GO:0005886 | plasma membrane |
| purple | 356 | 314 | 7 | 3.15E-05 | 0.53883 | 112 | 4053 | GO:0005576 | extracellular region |
| purple | 356 | 314 | 8 | 3.20E-05 | 0.54795 | 15 | 220 | GO:0019932 | second-messenger-mediated signaling |
| purple | 356 | 314 | 9 | 4.85E-05 | 0.83075 | 45 | 1240 | GO:0007155 | cell adhesion |
| purple | 356 | 314 | 10 | 4.91E-05 | 0.84023 | 52 | 1512 | GO:0042592 | homeostatic process |
| red | 779 | 649 | 1 | 7.85E-05 | 1 | 7 | 27 | GO:0007099 | centriole replication |
| red | 779 | 649 | 2 | 0.00013 | 1 | 7 | 29 | GO:0098534 | centriole assembly |
| red | 779 | 649 | 3 | 0.00033 | 1 | 5 | 16 | GO:0008392 | arachidonic acid epoxygenase activity |
| red | 779 | 649 | 4 | 0.00034 | 1 | 11 | 79 | GO:0000123 | histone acetyltransferase complex |
| red | 779 | 649 | 5 | 0.00059 | 1 | 9 | 59 | GO:0051298 | centrosome duplication |
| red | 779 | 649 | 6 | 0.00063 | 1 | 3 | 5 | GO:0061743 | motor learning |
| red | 779 | 649 | 7 | 0.00099 | 1 | 10 | 76 | GO:0007098 | centrosome cycle |
| red | 779 | 649 | 8 | 0.00103 | 1 | 5 | 20 | GO:0019373 | epoxygenase P450 pathway |
| red | 779 | 649 | 9 | 0.00123 | 1 | 3 | 6 | GO:1990316 | ATG1/ULK1 kinase complex |
| red | 779 | 649 | 10 | 0.00123 | 1 | 3 | 6 | GO:0003836 | beta-galactoside (CMP) alpha-2,3-sialyltransferase activity |
| royalblu | 129 | 75 | 1 | 0.00077 | 1 | 2 | 9 | GO:0018095 | protein polyglutamylation |
| royalblu | 129 | 75 | 2 | 0.00123 | 1 | 3 | 45 | GO:0032648 | regulation of interferon-beta production |
| royalblu | 129 | 75 | 3 | 0.0014 | 1 | 2 | 12 | GO:0007342 | fusion of sperm to egg plasma membrane |
| royalblu | 129 | 75 | 4 | 0.0014 | 1 | 2 | 12 | GO:0032688 | negative regulation of interferon-beta production |
| royalblu | 129 | 75 | 5 | 0.0014 | 1 | 3 | 47 | GO:0032608 | interferon-beta production |
| royalblu | 129 | 75 | 6 | 0.00218 | 1 | 4 | 116 | GO:0032479 | regulation of type I interferon production |
| royalblu | 129 | 75 | 7 | 0.00225 | 1 | 4 | 117 | GO:0032606 | type I interferon production |
| royalblu | 129 | 75 | 8 | 0.00471 | 1 | 1 | 1 | GO:0021813 | cell-cell adhesion in neuronal-glial interactions in cerebral cortex radial glia guided migration |
| royalblu | 129 | 75 | 9 | 0.00471 | 1 | 1 | 1 | GO:0005503 | all-trans retinal binding |
| royalblu | 129 | 75 | 10 | 0.00471 | 1 | 1 | 1 | GO:0015196 | L-tryptophan transmembrane transporter activity |

| | | | | | | | | | |
|---|---|---|---|---|---|---|---|---|---|
| salmon | 255 | 186 | 1 | 0.00014 | 1 | 2 | 2 | GO:0004458 | D-lactate dehydrogenase (cytochrome) activity |
| salmon | 255 | 186 | 2 | 0.00021 | 1 | 64 | 3641 | GO:0046872 | metal ion binding |
| salmon | 255 | 186 | 3 | 0.00036 | 1 | 16 | 515 | GO:0016569 | covalent chromatin modification |
| salmon | 255 | 186 | 4 | 0.00038 | 1 | 64 | 3719 | GO:0043169 | cation binding |
| salmon | 255 | 186 | 5 | 0.00039 | 1 | 14 | 416 | GO:0016570 | histone modification |
| salmon | 255 | 186 | 6 | 0.0004 | 1 | 2 | 3 | GO:0071001 | U4/U6 snRNP |
| salmon | 255 | 186 | 7 | 0.00063 | 1 | 4 | 34 | GO:0017069 | snRNA binding |
| salmon | 255 | 186 | 8 | 0.00064 | 1 | 3 | 15 | GO:0070816 | phosphorylation of RNA polymerase II C-terminal domain |
| salmon | 255 | 186 | 9 | 0.00132 | 1 | 3 | 19 | GO:0071425 | hematopoietic stem cell proliferation |
| salmon | 255 | 186 | 10 | 0.00132 | 1 | 2 | 5 | GO:0019323 | pentose catabolic process |
| tan | 261 | 198 | 1 | 1.89E-06 | 0.03238 | 3 | 3 | GO:0001156 | TFIIIC-class transcription factor binding |
| tan | 261 | 198 | 2 | 1.86E-05 | 0.31786 | 3 | 5 | GO:0001025 | RNA polymerase III transcription factor binding |
| tan | 261 | 198 | 3 | 0.00091 | 1 | 2 | 4 | GO:0001032 | RNA polymerase III type 3 promoter DNA binding |
| tan | 261 | 198 | 4 | 0.0015 | 1 | 2 | 5 | GO:0001030 | RNA polymerase III type 1 promoter DNA binding |
| tan | 261 | 198 | 5 | 0.0015 | 1 | 2 | 5 | GO:0001031 | RNA polymerase III type 2 promoter DNA binding |
| tan | 261 | 198 | 6 | 0.0031 | 1 | 2 | 7 | GO:0071233 | cellular response to leucine |
| tan | 261 | 198 | 7 | 0.0031 | 1 | 2 | 7 | GO:0031931 | TORC1 complex |
| tan | 261 | 198 | 8 | 0.0031 | 1 | 2 | 7 | GO:0070652 | HAUS complex |
| tan | 261 | 198 | 9 | 0.0031 | 1 | 2 | 7 | GO:0001016 | RNA polymerase III regulatory region DNA binding |
| tan | 261 | 198 | 10 | 0.0031 | 1 | 2 | 7 | GO:0008467 | [heparan sulfate]-glucosamine 3-sulfotransferase 1 activity |
| turquois | 3906 | 3148 | 1 | 1.49E-06 | 0.02558 | 151 | 538 | GO:0048812 | neuron projection morphogenesis |
| turquois | 3906 | 3148 | 2 | 1.59E-06 | 0.02722 | 140 | 492 | GO:0048667 | cell morphogenesis involved in neuron differentiation |
| turquois | 3906 | 3148 | 3 | 1.93E-06 | 0.03308 | 154 | 553 | GO:0048858 | cell projection morphogenesis |
| turquois | 3906 | 3148 | 4 | 3.20E-06 | 0.0548 | 288 | 1150 | GO:0030182 | neuron differentiation |
| turquois | 3906 | 3148 | 5 | 6.05E-06 | 0.10358 | 156 | 572 | GO:0032990 | cell part morphogenesis |
| turquois | 3906 | 3148 | 6 | 8.83E-06 | 0.15118 | 478 | 2045 | GO:0007399 | nervous system development |
| turquois | 3906 | 3148 | 7 | 9.01E-06 | 0.1543 | 115 | 401 | GO:0007409 | axonogenesis |
| turquois | 3906 | 3148 | 8 | 1.92E-05 | 0.32875 | 231 | 916 | GO:0048666 | neuron development |
| turquois | 3906 | 3148 | 9 | 3.78E-05 | 0.64645 | 83 | 279 | GO:0005261 | cation channel activity |
| turquois | 3906 | 3148 | 10 | 6.14E-05 | 1 | 213 | 850 | GO:0000902 | cell morphogenesis |
| yellow | 1731 | 1430 | 1 | 3.85E-08 | 0.00066 | 34 | 138 | GO:0052652 | cyclic purine nucleotide metabolic process |
| yellow | 1731 | 1430 | 2 | 4.66E-08 | 0.0008 | 34 | 139 | GO:0009190 | cyclic nucleotide biosynthetic process |
| yellow | 1731 | 1430 | 3 | 3.00E-07 | 0.00513 | 36 | 163 | GO:0007187 | G-protein coupled receptor signaling pathway, coupled to cyclic nucleotide second messenger |

| | | | | | | | | | |
|---|---|---|---|---|---|---|---|---|---|
| yellow | 1731 | 1430 | 4 | 5.16E-07 | 0.00883 | 23 | 82 | GO:0031279 | regulation of cyclase activity |
| yellow | 1731 | 1430 | 5 | 6.93E-07 | 0.01187 | 27 | 108 | GO:0006171 | cAMP biosynthetic process |
| yellow | 1731 | 1430 | 6 | 8.75E-07 | 0.01498 | 69 | 425 | GO:0004930 | G-protein coupled receptor activity |
| yellow | 1731 | 1430 | 7 | 1.64E-06 | 0.02804 | 26 | 106 | GO:0030817 | regulation of cAMP biosynthetic process |
| yellow | 1731 | 1430 | 8 | 1.83E-06 | 0.03138 | 109 | 781 | GO:0007186 | G-protein coupled receptor signaling pathway |
| yellow | 1731 | 1430 | 9 | 2.07E-06 | 0.0354 | 112 | 810 | GO:0004888 | transmembrane signaling receptor activity |
| yellow | 1731 | 1430 | 10 | 2.31E-06 | 0.03957 | 47 | 259 | GO:0034702 | ion channel complex |
